# Targeting Aquaporin-4 Subcellular Localization to Treat Central Nervous System Edema

**DOI:** 10.1016/j.cell.2020.03.037

**Published:** 2020-05-14

**Authors:** Philip Kitchen, Mootaz M. Salman, Andrea M. Halsey, Charlotte Clarke-Bland, Justin A. MacDonald, Hiroaki Ishida, Hans J. Vogel, Sharif Almutiri, Ann Logan, Stefan Kreida, Tamim Al-Jubair, Julie Winkel Missel, Pontus Gourdon, Susanna Törnroth-Horsefield, Matthew T. Conner, Zubair Ahmed, Alex C. Conner, Roslyn M. Bill

**Affiliations:** 1 School of Life & Health Sciences, Aston University, Aston Triangle, Birmingham B4 7ET, UK; 2 Department of Cell Biology, Harvard Medical School, Boston, MA 02115, USA; 3 Program in Cellular and Molecular Medicine, Boston Children’s Hospital, Boston, MA 02115, USA; 4 Department of Pharmacology, College of Pharmacy, University of Mosul, Mosul 41002, Iraq; 5 Neuroscience and Ophthalmology, Institute of Inflammation and Ageing, College of Medical and Dental Sciences, University of Birmingham, Edgbaston, Birmingham B15 2TT, UK; 6 Department of Biochemistry and Molecular Biology, Cumming School of Medicine, University of Calgary, Calgary, AB T2N 4Z6, Canada; 7 Department of Biological Sciences, University of Calgary, Calgary, AB T2N 4N1, Canada; 8 Department of Clinical Laboratory Science, College of Applied Medical Science, Shaqra University, Shaqra, Saudi Arabia; 9 Department of Biochemistry and Structural Biology, Lund University, PO Box 124, 221 00 Lund, Sweden; 10 Department of Biomedical Sciences, University of Copenhagen, 2200 Copenhagen, Denmark; 11 Department of Experimental Medical Science, Lund University, PO Box 118, 221 00 Lund, Sweden; 12 School of Sciences, Research Institute in Healthcare Science, University of Wolverhampton, Wolverhampton WV1 1LY, UK; 13 Institute of Clinical Sciences, College of Medical and Dental Sciences, University of Birmingham, Edgbaston, Birmingham B15 2TT, UK

**Keywords:** Aquaporin, AQP4, edema, astrocyte, spinal cord injury, traumatic brain injury, trifluoperazine, calmodulin, protein kinase A, TRPV4, oedema

## Abstract

Swelling of the brain or spinal cord (CNS edema) affects millions of people every year. All potential pharmacological interventions have failed in clinical trials, meaning that symptom management is the only treatment option. The water channel protein aquaporin-4 (AQP4) is expressed in astrocytes and mediates water flux across the blood-brain and blood-spinal cord barriers. Here we show that AQP4 cell-surface abundance increases in response to hypoxia-induced cell swelling in a calmodulin-dependent manner. Calmodulin directly binds the AQP4 carboxyl terminus, causing a specific conformational change and driving AQP4 cell-surface localization. Inhibition of calmodulin in a rat spinal cord injury model with the licensed drug trifluoperazine inhibited AQP4 localization to the blood-spinal cord barrier, ablated CNS edema, and led to accelerated functional recovery compared with untreated animals. We propose that targeting the mechanism of calmodulin-mediated cell-surface localization of AQP4 is a viable strategy for development of CNS edema therapies.

## Highlights

• Hypoxia triggers an increase in AQP4-mediated flux of water into astrocytes
• Translocation of AQP4 to the astrocyte cell surface drives increased water flux
• AQP4 cell-surface localization is mediated by a CaM- and PKA-dependent mechanism
• Inhibition of AQP4 localization with the licensed drug TFP halts CNS edema in rats

## Graphical Abstract

**Figure:**
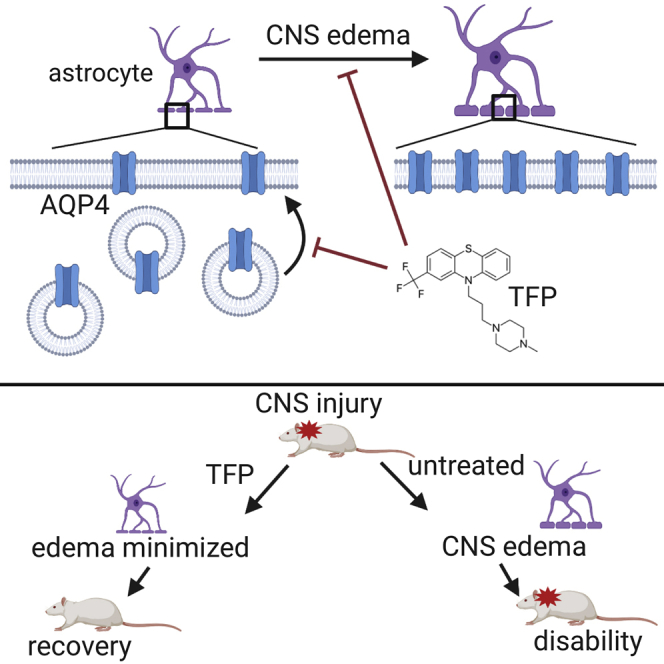

## Teaser

Modulating the subcellular localization of the water channel protein AQP4 may be a therapeutic option for treatment of brain and spinal cord edemas.

## Introduction

Swelling of the brain or spinal cord (known as central nervous system [CNS] edema) is a result of increased CNS water content and can occur after trauma, infection, tumor growth, or obstruction of blood supply (Jha et al., 2019, Liang et al., 2007). Traumatic injury and stroke are major causes; according to World Health Organization (WHO) data, worldwide each year, 60 million people sustain a traumatic brain or spinal cord injury (TBI or SCI), and 15 million people suffer a stroke (5 million die, another 5 million are permanently disabled). These injuries can be fatal or lead to long-term disability, psychiatric disorders, substance abuse, or self-harm (Fazel et al., 2014).

The pathophysiology of CNS edema is complex, being dependent on the nature of the initial injury and the specific response of the patient (Jha et al., 2019). Cytotoxic edema is the accumulation of water in intact cells. It is rapidly triggered when a state of hypoxia causes loss of energy-dependent solute homeostasis (Bordone et al., 2019). This drives water influx (Tang and Yang, 2016) down an osmotic gradient into perivascular astrocytes, causing them to swell (Rosenberg, 1999, Simard et al., 2007). The accumulation of intracellular water disrupts the local osmotic environment, resulting in ionic edema (Kawamata et al., 2007) and breakdown of the blood-brain barrier (BBB) or blood-spinal cord barrier (BSCB), which may already be damaged by the initial injury. Fluid accumulation in extracellular spaces across a compromised BBB/BSCB is known as vasogenic edema (Simard et al., 2007) and may persist for many days (Winkler et al., 2016). Cytotoxic and vasogenic edema are key interdependent contributors to the development of CNS edema, with extended periods of cytotoxic edema inducing vasogenic edema and vice versa (Jha et al., 2019).

Cytotoxic edema is the premorbid process of pathological cell swelling in SCI (Goodman et al., 1976, Rowland et al., 2008, Saadoun and Papadopoulos, 2010), TBI (Klatzo, 1987, Manley et al., 2004, Stokum et al., 2015), and stroke (Chu et al., 2016, Liang et al., 2007, Manley et al., 2004). Current therapies only manage the symptoms and include surgical intervention to make space for injured tissues to swell or treatment with hyperosmotic agents (Raslan and Bhardwaj, 2007). Crucially, however, such interventions have high risk and low efficacy and can be applied only after the damaging edema has fully developed (Gopalakrishnan et al., 2018, Torre-Healy et al., 2012). Treatments targeting the numerous pathways leading to CNS edema formation are likely to be more valuable than those aimed at removing the edema after it has formed (Jha et al., 2019). Consequently, there is a pressing and unmet clinical need for treatments that can stop CNS edema before it develops.

Regulation of astrocyte cell volume is central for avoiding the damaging consequences of CNS edema (Vella et al., 2015, Zhao et al., 2003). Aquaporins (AQPs) are plasma membrane channels that play an integral role in the development of cytotoxic edema because they facilitate bidirectional transmembrane water flow. AQP4 is the principal member of this family in the CNS (Papadopoulos and Verkman, 2013), being expressed abundantly in astrocytes (Oklinski et al., 2014, Oshio et al., 2004). In AQP4 knockout mice, post-ischemic brain edema was reduced by 35% compared with wild-type controls (Manley et al., 2000), whereas glial-conditional knockout mice showed a 31% reduction in brain water uptake after systemic hypo-osmotic stress (Haj-Yasein et al., 2011), strongly supporting AQP4 as a key player in cytotoxic edema. The role of AQP4 in SCI has also been examined in AQP4 knockout mice (Kimura et al., 2010, Saadoun et al., 2008). These and other studies suggest that AQP4 has different roles in development and resolution of CNS edema, with water flow through AQP4 driving cytotoxic edema development in the early post-injury stage but later clearing vasogenic edema. Consequently, reversible inhibition of AQP4 function during the acute phase is a viable strategy to prevent CNS edema (Verkman et al., 2017). However, despite intense efforts over many years, no water-channel-blocking drugs for any AQP have been approved for use in humans (Abir-Awan et al., 2019, Liang et al., 2007, Verkman et al., 2014).

We focused on a conceptually different approach: targeting AQP4 subcellular localization. We discovered previously that AQP4 cell-surface abundance is rapidly and reversibly regulated in response to changes in tonicity in primary cortical astrocytes (Kitchen et al., 2015, Salman et al., 2017a). Here we present the protein kinase A (PKA) and calmodulin (CaM) dependence of AQP4 subcellular localization in astrocytes. We show that acute hypoxia leads to subcellular relocalization of AQP4 in primary cortical astrocytes and that this is accompanied by increased membrane water permeability. Direct interaction between AQP4 and CaM causes a specific conformational change in the carboxyl terminus of AQP4 and drives AQP4 cell-surface localization. Using a rat SCI model of CNS edema, we present *in vivo* evidence that inhibitors of AQP4 subcellular localization to the BSCB reduce spinal cord water content following CNS injury. All measured pathophysiological features of SCI are counteracted by pharmacological inhibition of CaM or PKA. Using trifluoperazine (TFP), a CaM antagonist that is approved as an antipsychotic by the US Food and Drug Administration and the UK National Institute for Health and Care Excellence (NICE, 2019), we found a protective effect against the sensory and locomotor deficits following SCI. Treated rats recovered in 2 weeks compared with untreated animals that still showed functional deficits after 6 weeks. Our findings reveal that targeting AQP4 subcellular localization following CNS injury has profound effects on the extent of subsequent damage and recovery. To our knowledge, an effective AQP4-targeted intervention, which has major implications for the future treatment of CNS edema, has not been demonstrated previously. Overall, we show that targeting the mechanism of CaM-mediated AQP4 subcellular relocalization is a viable strategy for development of CNS edema therapies. This has implications for the development of new approaches to treat a wide range of neurological conditions.

## Results

### Hypoxia Induces AQP4 Subcellular Localization *In Vitro*

**Figure 1:**
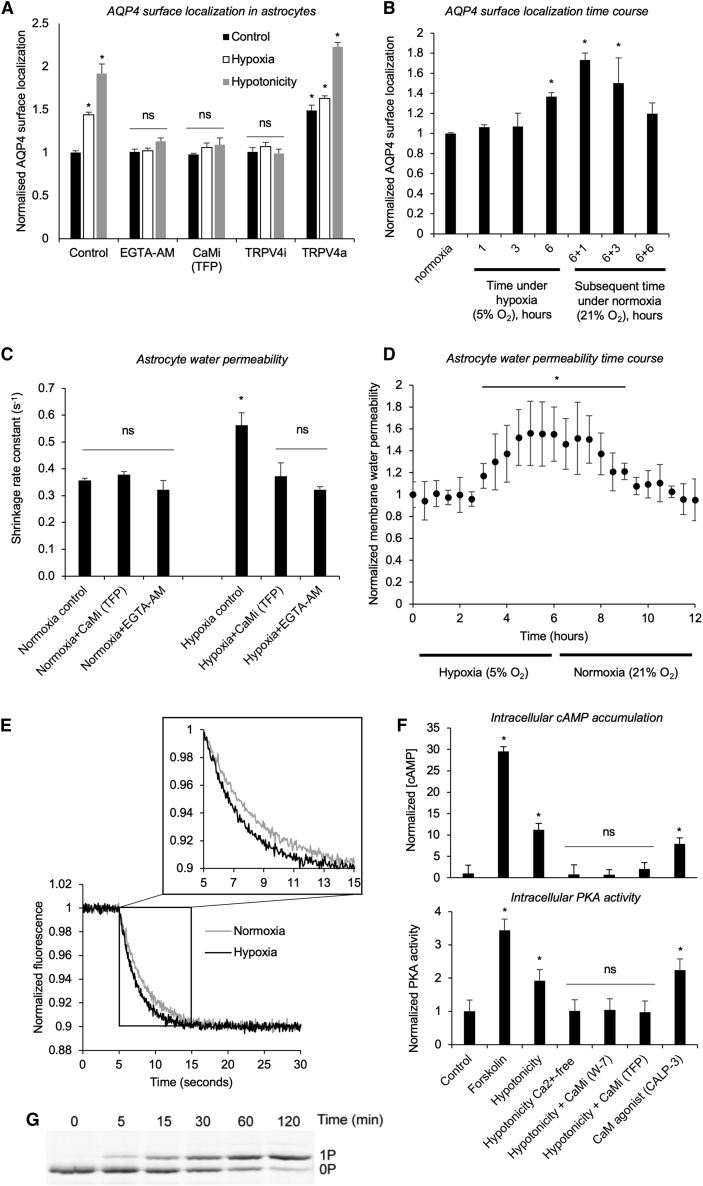
Hypoxia Induces AQP4 Subcellular Relocalization in Primary Cortical Astrocytes (A) Mean fold change in AQP4 surface expression (±SEM), measured by cell-surface biotinylation in primary cortical astrocytes. Cells were treated with 5% oxygen for 6 h (hypoxia) or 85 mOsm/kg H_2_O (hypotonicity) compared with untreated normoxic astrocytes (control). The CaM inhibitor (CaM_i_) was 127 μM trifluoperazine (TFP). The TRPV4 inhibitor (TRPV4_i_) was 4.8 μM HC-067047, and the intracellular Ca^2+^ chelator was 5 μM EGTA-AM. The TRPV4 channel agonist (TRPV4_a_) was 2.1 μM GSK1016790A. Kruskal-Wallis with Conover-Inman post hoc tests were used to identify significant differences between samples. ^∗^p < 0.05; ns represents p > 0.05 compared with the untreated control (Table S2; n = 4). (B) Mean fold change in AQP4 surface expression (±SEM) with time under hypoxia. Rat primary cortical astrocytes were exposed to 5% oxygen, and AQP4 surface expression was measured by cell-surface biotinylation after 1, 3, and 6 h and compared with untreated normoxic astrocytes (normoxia). Cells were returned to normoxic conditions (21% oxygen), and AQP4 surface expression was measured at 1, 3, and 6 h. ^∗^p < 0.05 by ANOVA followed by t test with Bonferroni correction (Table S2; n = 3). (C) Calcein fluorescence quenching in response to elevation of extracellular osmolality to 600 mOsm with mannitol was used to quantify astrocyte plasma membrane water permeability following the same hypoxia and inhibitor regimen described in (A). ^∗^p < 0.05; ns indicates p > 0.05 by ANOVA followed by t test with Bonferroni correction (Table S2; n = 3). (D) Normalized membrane water permeability of hypoxic rat primary cortical astrocytes over 6 h. Cells were returned to normoxic conditions (21% oxygen), and permeability was measured over the subsequent 6 h. ^∗^p < 0.05 compared with the t = 0 normoxic control (Table S2; n = 3). (E) Representative calcein fluorescence quenching traces for hypoxia and normoxia. (F) Intracellular cAMP accumulation in rat primary cortical astrocytes (±SEM) in response to forskolin, hypotonicity (with or without extracellular Ca^2+^ [the intracellular Ca^2+^ chelator was 5 μM EGTA-AM] or the CaM_i_ W-7 or TFP), and the CaM_a_ peptide CALP-3. PKA activity in lysates made from rat astrocytes subjected to the same treatments. Activity is normalized to the average of the untreated control. ^∗^p < 0.05. ns represents p > 0.05 by ANOVA followed by t test with Bonferroni correction (Table S2; n = 3). (G) Phosphorylation of glutathione S-transferase (GST)-recAQP4ct, analyzed by Phos-tag SDS-PAGE. The phosphorylation stoichiometry was judged to be complete (∼1 mol phosphate/mol GST-recAQP4ct, 1P) after 2 h.

**Figure S1:**
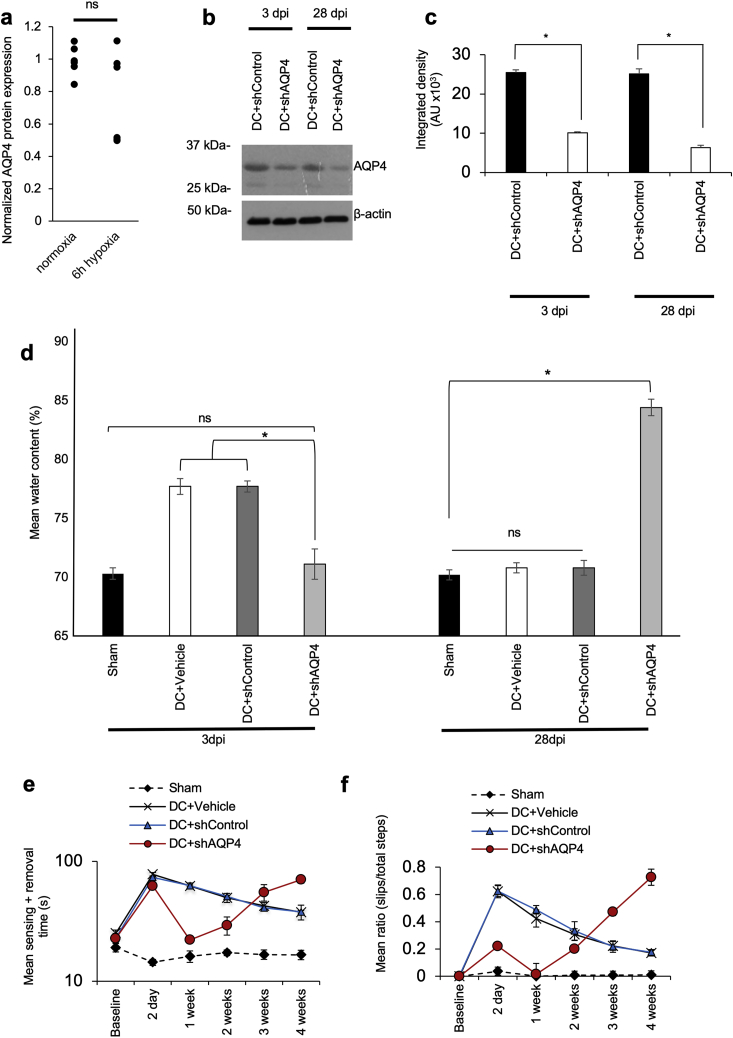
Knockdown of AQP4 Suppresses Acute Cytotoxic Edema and Improves Functional Recovery in Rats at 3 dpi but Not at 28 dpi following DC Crush Injury a, ELISA for AQP4 showing no increase in total AQP4 protein in primary astrocytes after 6 h under hypoxia (5% O_2_; n = 6); b, Representative immunoblot and c, densitometry to confirm 60% and 75% knockdown of AQP4 using *in vivo* JetPEI-delivered shRNA to AQP4 (shAQP4) at 3 dpi and 28 dpi following DC crush injury (n = 3 independent repeats from 3 pooled rat spinal cords/experiment (total n = 9 rats/condition)); d, Spinal cord water content was significantly suppressed at 3 dpi, but at 28 dpi the water content in DC + shAQP4-treated animals was significantly higher than DC + Vehicle-treated controls, despite 75% AQP4 protein knockdown (n = 3-4 rats/condition, 3 independent repeats (total n = 10 rats/condition)); e, Knockdown of AQP4 improved tape sensing and removal time up to 1 week after DC crush injury, but the sensing and removal time gradually increased to above DC + vehicle-treated controls at 4 weeks after DC crush injury (n = 3-4 rats/condition, 3 independent repeats (total n = 10 rats/condition)); f, The early improvement in ladder crossing ability of rats gradually worsened 1 week after and at 28dpi is higher than DC+vehicle-treated controls (n = 3-4 rats/condition, 3 independent repeats (total n = 10 rats/condition)). ^∗^ represents p < 0.05, ns represents p > 0.05 (see Table S2 for p values). Animals were euthanized at 28dpi due to episodes of vomiting, gait problems, lethargy and convulsions, possibly reflecting their inability to regulate water in the CNS. Related to Figure 2.

The relative increase in tonicity that leads to CNS edema can be modeled by hypotonic treatment or hypoxic exposure of primary astrocytes (Orellana et al., 2010, Slovinska et al., 2016, Yu et al., 1989). To date, we have used a hypotonicity model to study AQP4 translocation (Kitchen et al., 2015, Salman et al., 2017a). Here we subjected primary astrocytes to both stimuli to show that tonicity-triggered AQP4 subcellular relocalization can be recapitulated *in vitro* by treating primary cortical astrocytes with 5% oxygen for 6 h (hypoxia) (Figure 1A). The same inhibitors have similar effects in hypoxic and hypotonic models (Figure 1A). Chelation of Ca^2+^ or CaM inhibition through EGTA-AM or TFP, respectively, reduced AQP4 translocation to control levels following hypoxic or hypotonic treatment (Figure 1A). When normoxic primary cortical astrocytes were treated with 5% oxygen, AQP4 cell-surface abundance increased over 6 h of hypoxia compared with untreated normoxic astrocytes (Figure 1B). There was no increase in the total amount of AQP4 protein (Figure S1A). A return to normoxic conditions (21% oxygen) reduced AQP4 cell-surface abundance over the subsequent 6 h (Figure 1B). Calcein fluorescence quenching was used to quantify astrocyte plasma membrane water permeability following hypoxia and inhibitor treatment (Figure 1C). The increase in shrinkage rate constant for human primary cortical astrocytes treated with 5% oxygen for 6 h (hypoxia) compared with controls mirrored the increase seen in AQP4 surface localization in the same cells (Figure 1A). This increase was ablated by chelation of Ca^2+^ or CaM inhibition through EGTA-AM or TFP, respectively. The increase in AQP4 cell-surface localization (Figure 1B) was mirrored by an increase in normalized membrane water permeability and its subsequent decay following restoration of normoxia (Figure 1D). Representative calcein fluorescence quenching traces are shown in Figure 1E. These results demonstrate that hypoxia induces AQP4 subcellular relocalization, leading to an increase in astrocyte membrane water permeability.

The transient receptor potential vanilloid 4 (TRPV4) channel is linked to the pathology of edema (Hoshi et al., 2018) and is known to be activated by cell swelling (Heller and O’Neil, 2007). We therefore inhibited TRPV4 following treatment with 5% oxygen for 6 h (hypoxia) or 85 mOsm/kg H_2_O (hypotonicity) and compared AQP4 cell-surface biotinylation in human primary cortical astrocytes with data for untreated normoxic astrocytes (control). Inhibition of TRPV4 with HC-067047 (TRPV4_i_) ablated AQP4 translocation (Figure 1A). Agonism of TRPV4 with GSK1016790A (TRPV4_a_) increased AQP4 surface translocation in the hypotonicity and hypoxia models (Figure 1A) and induced AQP4 translocation in the absence of either trigger, suggesting that Ca^2+^ influx through TRPV4 is sufficient to activate AQP4 translocation. This established the role of TRPV4 in regulatory AQP4 translocation.

### CaM and PKA Have Central Roles in AQP4 Subcellular Relocalization

We previously demonstrated that inhibiting CaM (with TFP or W-7) or the action of PKA (with H89, myr-PKI 14-22 amide or via mutagenesis of AQP4-S276) reduced hypotonicity-induced subcellular relocalization of AQP4 in HEK293 cells (Kitchen et al., 2015). We next investigated the role of CaM in PKA activation by measuring the induction of CaM-dependent cyclic AMP (cAMP) accumulation following a hypotonic trigger. cAMP levels increased approximately 10-fold upon hypotonic treatment (Figure 1F), and this was ablated by CaM inhibition by TFP or W-7. The same ablation was seen following removal of extracellular Ca^2+^ from the culture medium. The CaM agonist CALP-3 (Villain et al., 2000) recapitulated the effects of hypotonicity on cAMP accumulation. Figure 1F shows that PKA activity correlated with cAMP concentration in primary rat astrocyte cell lysates under all of the conditions described above. This demonstrates that Ca^2+^-dependent activation of CaM in astrocytes triggers activation of PKA. *In vitro* phosphorylation of the recombinant AQP4 carboxyl terminus (recAQP4ct; Figure 1G) demonstrated a single PKA phosphorylation site.

### AQP4-Dependent CNS Edema Is Ablated by Inhibition of CaM or PKA *In Vivo*

To investigate CNS edema *in vivo*, we used a rat SCI model consisting of dorsal column (DC) crush injury at the T8 vertebra (Almutiri et al., 2018, Lagord et al., 2002, Surey et al., 2014) and measured spinal cord water content 3, 7, and 28 days post-injury (dpi). To confirm that development of CNS edema is AQP4 dependent, plasmids encoding AQP4 shRNA were injected into the lesion site at the time of injury. AQP4 knockdown (Figures S1B and S1C) resulted in reduced spinal cord water content at 3 dpi (Figure S1D) and recovery of sensory and locomotor function within 1 week compared with injured animals (DC+vehicle) or shRNA controls (DC+shControl), which had elevated spinal water content at 3 dpi and were still functionally impaired at 4 weeks (Figures S1E and S1F). Notably, at 28 dpi, water content in the shAQP4 group was elevated above that of control animals (sham, DC+vehicle, DC+shControl). Following initial recovery within 1 week post-injury, behavioral function in the shAQP4 group deteriorated until the end of the experiment at 4 weeks (Figures S1E and S1F). The impaired ability of animals in the shAQP4 group to clear CNS edema is consistent with previous observations in AQP4-null mice (Papadopoulos and Verkman, 2007) and suggests that reversible blockade of AQP4 might be beneficial within 1 week post-injury.

**Figure 2:**
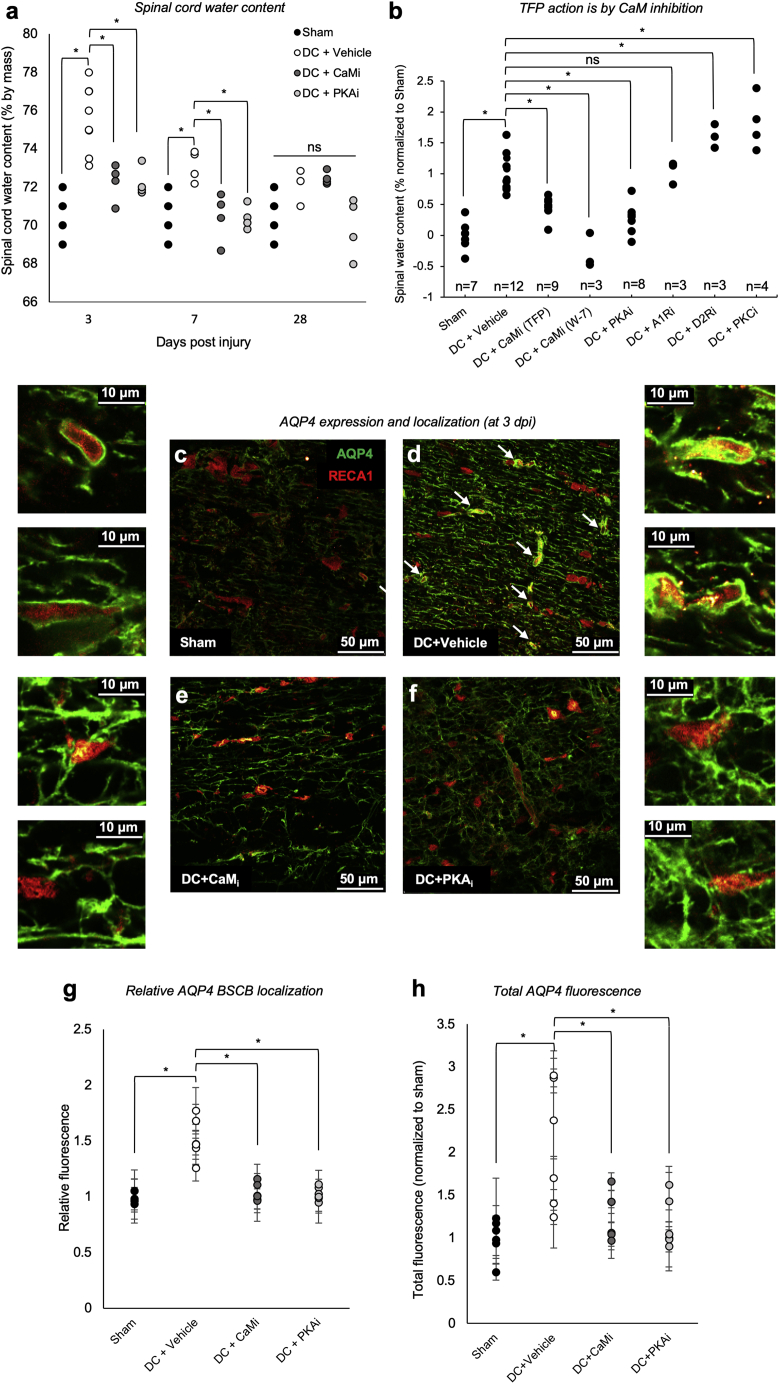
Inhibition of CaM or PKA Reduces Spinal Cord Water Content, AQP4 Translocation to the BSCB, and AQP4 Expression after DC Crush Injury *In Vivo* (A) Water content of the thoracic spinal cord 3 days after dorsal column (DC) crush and treatment with CaM or PKA inhibitors (CaM_i_ or PKA_i_, respectively). Sham, laminectomy only; DC+vehicle, T8 DC crush +PBS; DC+CaM_i_, DC crush + intra-lesion injection of 41 mM TFP; DC+PKA_i_, DC crush + intra-lesion injection of 10 μM H89; n = 4 rats per treatment group, except DC+vehicle 3 dpi (n = 8) and DC+vehicle 28 dpi (n = 3). (B) Water content of the thoracic spinal cord 3 days after DC crush to investigate the mode of action of TFP. DC+CaM_i_, DC crush + intra-lesion injection of 41 mM TFP or 164 mM W-7; DC+A1R_i_, DC crush + intra-lesion injection of 53 mM α1 adrenergic receptor antagonist terazosin; DC+D2Ri_i_, DC crush + intra-lesion injection of 6.6 mM dopamine D_2_ receptor antagonist L-741,626; DC+PKC_i_, DC crush + intra-lesion injection of 9.94 μM PKC inhibitor Gö 6983. n = 3–12 rats per treatment group, normalized to sham controls across multiple experiments. (C–F) Representative images used to quantify data in (G) and (H) (scale bar, 50 μm). Sham tissue (C) was compared with tissue from animals 3 days after DC crush (3 dpi; D), for which an increase in total AQP4 expression and translocation to the blood-spinal cord barrier (BSCB; white arrows identify astrocyte endfeet) is observed, which is ablated by treatment with TFP (E) or treatment with H89 (F). Two representative magnified images are shown for each panel. The contrast in the insets was manually adjusted to aid the eye (scale bars, 10 μm). (G) Quantification of at least 24 images (a minimum of 4 images per animal, n = 6) shows changes in AQP4 BSCB localization. (H) Quantification of at least 18 images (a minimum of 3 images per animal, n = 6) shows changes in total AQP4 expression. For all comparisons, ANOVA was followed by post hoc Bonferroni-corrected t tests. ^∗^p < 0.05, ns represents p > 0.05 (Table S2). See also Figure S1, Figure S2, Figure S3.

Figure 2A shows that CaM inhibition (CaM_i_; with TFP) or PKA inhibition (PKA_i_; with H89) attenuated DC crush injury-induced CNS edema when we compared spinal cord water content in uninjured rats (sham) with those receiving a DC crush injury (Almutiri et al., 2018, Lagord et al., 2002, Surey et al., 2014) immediately followed by treatment with direct injection of TFP or H89. A single dose was given, with the goal of acute inhibition of AQP4 subcellular relocalization. In agreement with literature values (Li and Tator, 1999), DC crush injury significantly increased mean spinal cord water content from 70.6% to 75.6% at 3 dpi, to 73.1% at 7 dpi, and returning to near control (sham) levels by 28 dpi. CaM_i_ with TFP effectively attenuated injury-induced elevation in spinal cord water content to 72.8% at 3 dpi and completely ablated it to 70.4% at 7 dpi (Figures 2A). PKA_i_ with H89 attenuated injury-induced rises in spinal cord water content to 71.5% at 3 dpi and 70.4% at 7 dpi (Figure 2A).

**Figure S2:**
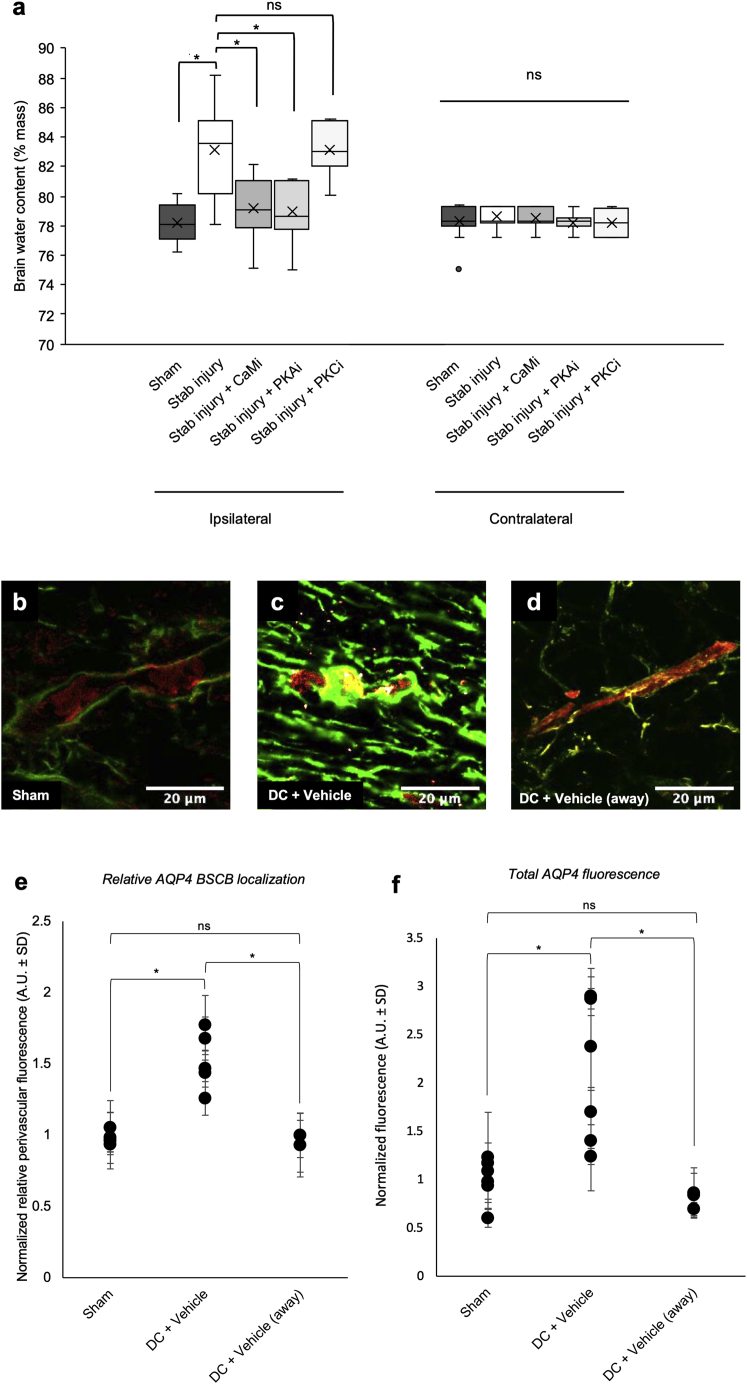
Targeted Inhibition of CaM or PKA Reduces Acute Brain Water Content after an *In Vivo* Brain Stab Injury Model of Cytotoxic Edema; Increases in AQP4 Expression and AQP4 Translocation to the Blood-Spinal-Cord Barrier are Localized to the Injury Site *In Vivo*. a, Water content of the ipsilateral and contralateral brain cortex 3 days after stab injury and treatment with CaM, PKA or PKC inhibitors (CaM_i_ or PKA_i_). Sham = craniotomy only; Stab injury+Vehicle = 3 mm cortical stab injury + PBS; Stab injury+CaM_i_ = 3 mm cortical stab injury + intra-lesion injection of 41 mM trifluoperazine (TFP); Stab injury + PKAi = 3 mm cortical stab injury + intra-lesion injection of 10 mM H89; Stab injury+PKCi = 3 mm cortical stab injury + intra-lesion injection of 9.94 μM Gö 6983; n = 18 rats per treatment group, ^∗^ represents p < 0.05, ns represents p > 0.05 (see Table S2 for p values). Central bars represent median, crosses represent the mean, outer bars represent upper and lower quartiles. Outliers (data points more than 1.5 × IQR from the median) are shown as separate points; b, Increases in AQP4 expression and AQP4 translocation to the blood-spinal-cord barrier are localized to the injury site *in vivo*. Representative images showing AQP4 expression (green) with endothelial cells labeled with RECA1 (red) in Sham animals; c, 3 days after DC crush (3 dpi), an increase in total AQP4 expression and translocation to the blood-spinal-cord barrier (BSCB) is observed at the injury site; d, At a minimum of 3 mm away from the lesion, total AQP4 expression and translocation to the BSCB are indistinguishable from that of sham animals; e, Normalized relative perivascular fluorescence (A.U. ± SD 3 days after DC crush injury; sham = laminectomy only; DC+vehicle = T8 DC crush + PBS at the injury site; DC+vehicle (away) = T8 DC crush + PBS at sites at a minimum of 3 mm away from the lesion site; f, Normalized total AQP4 fluorescence (A.U. ± SD) following the same experimental conditions described in panel e. ^∗^ represents p < 0.05, ns represents p > 0.05 (see Table S2 for p values). Related to Figure 2.

TFP is an antipsychotic and anti-anxiety medication; its effects are attributed to TFP antagonism of the dopamine D2 and α1 adrenergic receptors (Creese et al., 1996, Huerta-Bahena et al., 1983). To determine whether the effects of TFP were primarily through inhibition of CaM, we measured spinal cord water content at 3 dpi following treatment with the alternative CaM inhibitor W-7, the D2 antagonist L-741,626, and the α1 antagonist terazosin (Figure 2B). W-7 has no reported activity at the dopamine D2 or α1 adrenergic receptors. Although W-7 treatment recapitulated the effect of TFP on spinal cord water content the α1 antagonist did not. D2 and protein kinase C (PKC) inhibitors increased spinal cord water content (Figure 2B). These results demonstrate that inhibition of CaM or PKA attenuates DC crush injury-induced edema. Similar changes in water content were also observed in a cortical brain stab injury model following treatment with TFP. The stab injury-induced rise in water content 24 h post-injury was almost completely ablated by treatment with TFP (Figure S2A). Because TFP and H89 are inhibitors of AQP4 translocation *in vitro* (Kitchen et al., 2015), we used confocal microscopy to examine their effect on AQP4 translocation in astrocytes after DC crush injury *in vivo*.

### CNS Edema Is Associated with Increases in Total AQP4 Expression and AQP4 Subcellular Translocation to the BSCB; Both Are Ablated by CaM_i_ or PKA_i_

AQP4 expression levels increase following traumatic injury, including SCI (Nesic et al., 2006). In response to hypotonicity, AQP4 is translocated to the plasma membrane of primary rat and human astrocytes *in vitro* (Kitchen et al., 2015, Salman et al., 2017a), but this translocation has not been demonstrated *in vivo*. Rats were therefore subjected to a DC crush injury at T8 and treated with CaM inhibitor (TFP), PKA inhibitor (H89), or vehicle (PBS). Representative images used for quantification are shown in Figures 2C–2F. In Figure 2D, white arrows identify astrocyte processes physically associated with endothelia, which were assigned as astrocyte endfeet. Relative expression was calculated by dividing the fluorescence intensity of peri-endothelial AQP4 by the fluorescence intensity of non-endothelial-cell-associated AQP4. At 3 dpi, increases in AQP4 translocation to the BSCB (p = 3.2 × 10^−7^; Figure 2G) and total AQP4 levels (p = 0.002; Figure 2H) were observed in injured rats (DC+vehicle) compared with sham controls. Notably, 3 mm away from the injury site, increases in AQP4 translocation to the BSCB and total AQP4 levels were indistinguishable from the levels observed in sham controls (Figures S2B–S2F). Increased AQP4 localization to the BSCB and total AQP4 expression were ablated by treatment with the CaM inhibitor TFP or the PKA inhibitor H89. Peri-endothelial localization of AQP4 is consistent with its role in driving cytotoxic CNS edema (Stokum et al., 2015, Tang and Yang, 2016). Inhibition of increased AQP4 peri-endothelial localization was accompanied by a reduction in spinal cord water content (Figure 2A).

**Figure S3:**
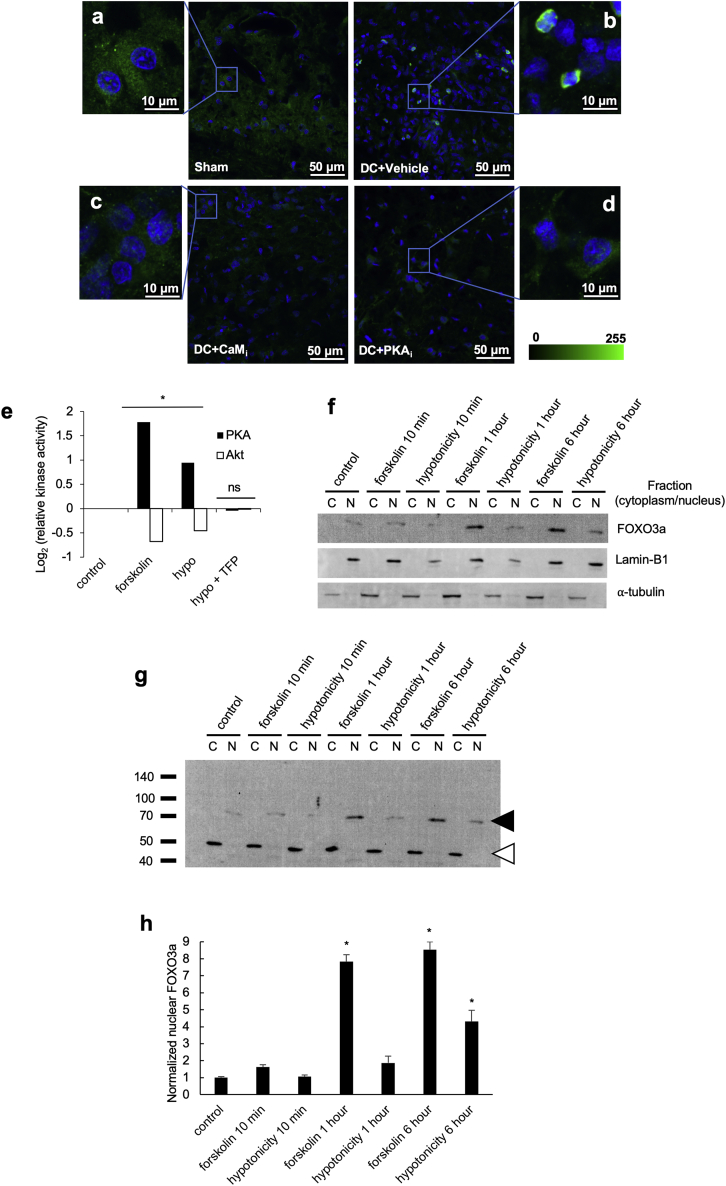
Subcellular Fractionation of Primary Rat Astrocytes Reveals Foxo3a Nuclear Translocation a-d, Immunofluorescence micrographs of rat spinal cord tissue stained for Foxo3a (green) and DNA (DAPI, blue) 3 days after dorsal column (DC) crush and treatment with PBS (DC + vehicle), TFP (DC + CaM_i_) or H89 (DC + PKA_i_) injected into the lesion site; zoomed-in images are shown for each panel; e, Relative protein kinase activity in cultured primary rat astrocytes subjected to PKA activation with forskolin (10^−5^ M) or 10 minutes extracellular hypotonicity (85 mOsm) in the absence or presence of TFP. Data (n = 3) are normalized to untreated controls. ^∗^ represents p < 0.05, ns represents p > 0.05 compared to untreated control by ANOVA followed by t test with Bonferroni correction, see Table S2 for p values); f, Immunoblot of fractionated primary rat astrocytes showing the abundance of Foxo3a, which is higher in the nuclear fraction (only degradation products are seen the cytoplasmic fraction as shown in panel g) following PKA activation with forskolin or hypotonicity. This is consistent with the image in panel b showing localization of Foxo3a to nuclei in injured spinal cord tissue. C = cytoplasmic fraction, N = nuclear fraction; g, Primary rat astrocytes were subjected to subcellular fractionation following activation of PKA by forskolin or hypotonicity. Intact Foxo3a was detected only in the nucleus (predicted molecular weight 71 kDa, black arrowhead). Degraded Foxo3a was detected in the cytoplasm (white arrowhead). h, Densitometry of nuclear Foxo3a signals shown in panel g and normalized to nuclear Lamin B. ^∗^ represents p < 0.05, ns represents p > 0.05 (see Table S2 for p values). Related to Figure 2.

Inhibition of AQP4 expression following CaM_i_ or PKA_i_ has not been reported previously *in vivo*. It is known that the forkhead transcription factor Foxo3a transcriptionally upregulates AQP4 after TBI in mice (Kapoor et al., 2013). Immunofluorescence micrographs of rat spinal cord tissue stained for Foxo3a (green) and DNA (DAPI, blue) 3 dpi showed nuclear localization of Foxo3a (DC+vehicle) in contrast to cytoplasmic localization in control (sham) tissue (Figures S3A and S3B) and consistent with increased AQP4 expression (Figure 2H). Treatment with TFP (DC+CaM_i_) or H89 (DC+PKA_i_) injected into the lesion site resulted in cytoplasmic localization of Foxo3a (Figures S3C and S3D). Foxo3a is well known to be regulated by phosphorylation through the action of Akt, with phosphorylation inhibiting the nuclear translocation of Foxo3a (Stefanetti et al., 2018). Akt PK activity was therefore measured by ELISA in cultured primary rat astrocytes subjected to PKA activation with forskolin (10^−5^ M) or 10-min extracellular hypotonicity (85 mOsm) in the absence or presence of TFP (Figure S3E). Akt activity was reduced following forskolin treatment, suggesting PKA-mediated inhibition of Akt activity in astrocytes. Following a hypotonic stimulus, the presence of TFP prevented activation of PKA activity and subsequent inhibition of Akt activity, consistent with ablation of Foxo3a translocation to the nucleus (Figures S3C and S3D). An immunoblot of fractionated primary rat astrocytes showed that the abundance of Foxo3a was higher in the nuclear fraction (only degradation products are seen the cytoplasmic fraction; Figure S3G) following PKA activation with forskolin or hypotonicity (Figures S3G and S3H). These data are consistent with localization of Foxo3a to nuclei in injured spinal cord tissue (Figure S3B).

### CaM Binds AQP4 Directly and the Interaction Is Inhibited by TFP

**Figure S4:**
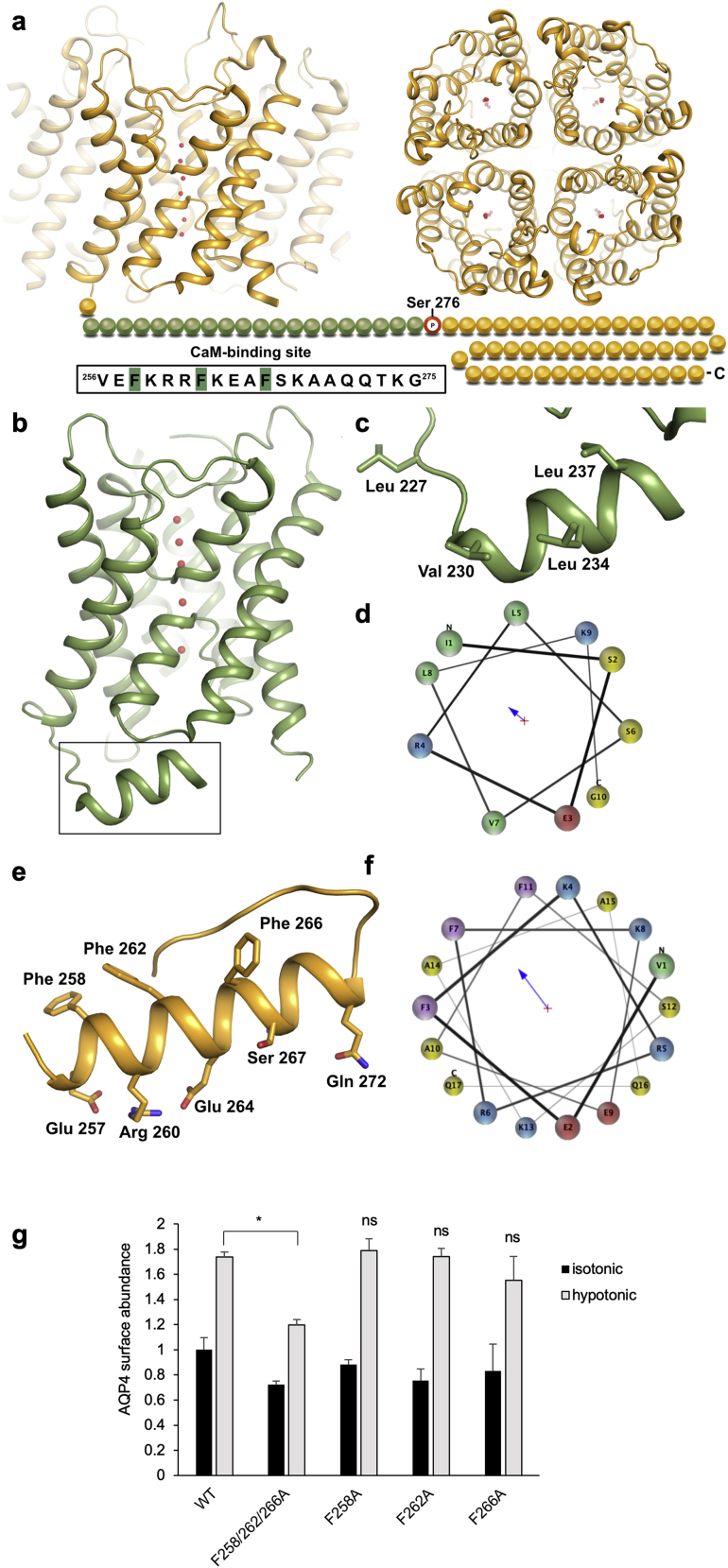
The Predicted AQP4 CBD and Its Comparison with the CBD in the AQP0 Crystal Structure a, Crystal structure of the human AQP4 tetramer viewed from the side of the membrane and from the extracellular side. The carboxyl terminus, for which there is no structural information, is shown as beads. The sequence of the predicted CaM-binding domain (green beads) is shown in the box with hydrophobic residues highlighted in green. The phosphorylation site at Ser 276 is highlighted with a red circle; b, Crystal structure of bovine AQP0 (PDB code 1YMG) showing the carboxy-terminal helix (black box) which harbors the CaM-binding domain of AQP0; c, Zoom-in on the CaM binding domain with residues involved in binding shown in stick representation; d, Helix wheel representation of the AQP0 carboxy-terminal helix showing its amphipathic character. Colors indicate residue types as follows: hydrophobic-green, basic-blue, acidic-red and polar-yellow; e, Top scoring structural model of the predicted AQP4 CaM-binding site generated by PEP-FOLD3. Hydrophobic and charged/polar residues on either side of the predicted helix are shown in stick representation; f, Helical wheel representation of the predicted helix in panel e showing its amphipathic character. Colors indicate residue types as follows: aromatic-purple, hydrophobic-green, basic-blue, acidic-red and polar-yellow; g, Hypotonicity-induced translocation of AQP4 in HEK293 cells is abrogated by a triple F258/262/266A mutation, but not by the corresponding single point mutations. ^∗^p < 0.05 by ANOVA followed by Bonferroni-corrected t test, ns denotes p > 0.05 (see Table S2 for p values). Related to Figure 3.

We have previously demonstrated that phosphorylation at S276 is necessary but not sufficient for AQP4 subcellular relocalization. This suggests that CaM has an additional role, independent of PKA, such as direct AQP4 binding, which has been reported for other AQPs (Rabaud et al., 2009, Reichow et al., 2013). Using the Calmodulin Target Database (Yap et al., 2000), we identified a putative CaM-binding site between residues 256 and 275. There is currently no structural information for this proximal part of the AQP4 carboxyl terminus immediately following the last transmembrane helix (Hiroaki et al., 2006, Ho et al., 2009, Tani et al., 2009) (Figure S4A). In the crystal structures of other mammalian AQPs (Frick et al., 2014, Gonen et al., 2004, Harries et al., 2004, Horsefield et al., 2008, Sui et al., 2001), this region forms an amphipathic helix that may be a common site for AQP regulatory protein-protein interactions (Kreida and Törnroth-Horsefield, 2015). For AQP0, the carboxy-terminal helix harbors a CaM-binding domain (CBD) involved in channel gating (Reichow et al., 2013, Reichow and Gonen, 2008; Figures S4B–S4D). A structural prediction of the proximal region of the AQP4 carboxyl terminus using PEP-FOLD3 (Lamiable et al., 2016) shows a similar amphipathic helix (Figures S4E and S4F). Three phenylalanine residues form a hydrophobic surface, with charged and polar residues on the other side, which is a typical characteristic of CaM-binding helices (Tidow and Nissen, 2013).

**Figure S5:**
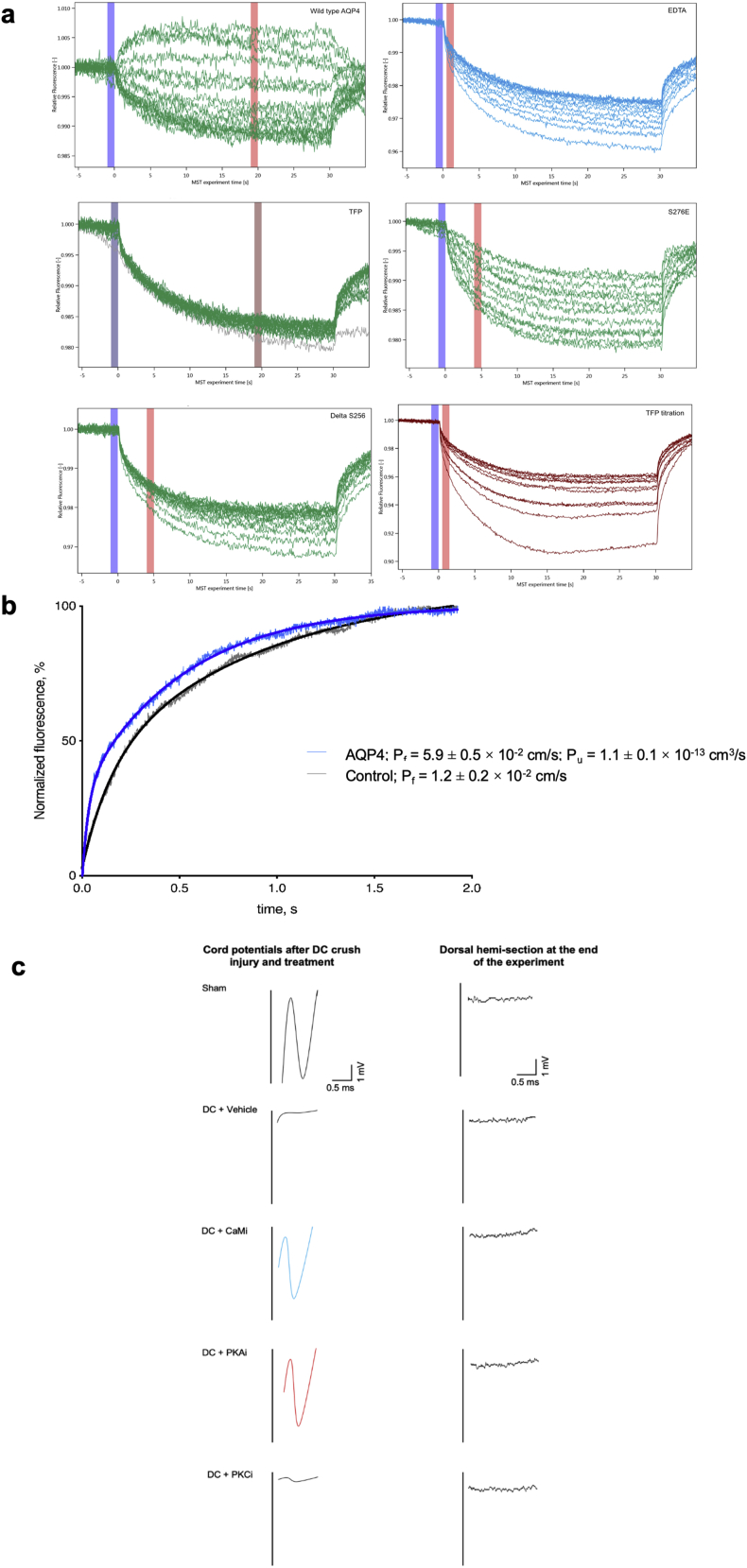
MST and CAP Experimental Controls a, Typical MST traces. The relative fluorescence is plotted against the experiment time. Each trace corresponds to a sample with a different concentration of AQP4 whereas calmodulin (CaM) concentration remained constant, except for the trifluoperazine (TFP) titration experiment (bottom right) in which TFP was varied and both AQP4 and CaM were kept constant. The difference in relative fluorescence before (blue column) and after (red column) heating is used to calculate ΔF_norm_. The position of the red column was determined by the M.O. Affinity Analysis software (Nanotemper) as the time interval that gave the best signal to noise ratio; b, Recombinant AQP4 is a functional water channel. Fluorescence traces from a proteoliposome shrinking assay showing that liposomes containing purified AQP4 (blue) have significantly higher water permeability than empty liposomes (gray). The increase in fluorescence corresponds to the fluorophore ((5)6-carboxyfluorescein) present on the inside of the liposomes becoming more fluorescent as the liposomes shrinks when mixed with hyperosmotic solution. The data were fitted to a double exponential function (solid blue and black lines for AQP4-containing and empty liposomes, respectively) and the rate constant (k) was used to calculate the osmotic water permeability (P_f_). For AQP4-containing liposomes P_f_ = 5.9 ± 0.5 × 10^−2^ cm/s and for control liposomes P_f_ = 1.2 ± 0.2 × 10^−2^ cm/s, corresponding to an AQP4 single channel permeability of 1.1 ± 0.1 × 10^−13^ cm^3^/s. Analysis by t test showed a statistically-significant difference between AQP4-containing liposomes and empty liposomes (p = 0.0015; Table S2). Related to Figure 3; c, Representative Spike 2 software processed CAP traces. A normal short-latency negative-positive CAP trace was observed in sham animals which was ablated in DC+vehicle- and DC+PKCi-treated rats, but partially restored in DC + CaMi and DC + PKAi-treated rats. Following a dorsal hemi-section in the same animals at the end of the experiment, CAP traces were ablated in all animals, indicating DC axon regeneration. Related to Figure 4.

**Figure 3:**
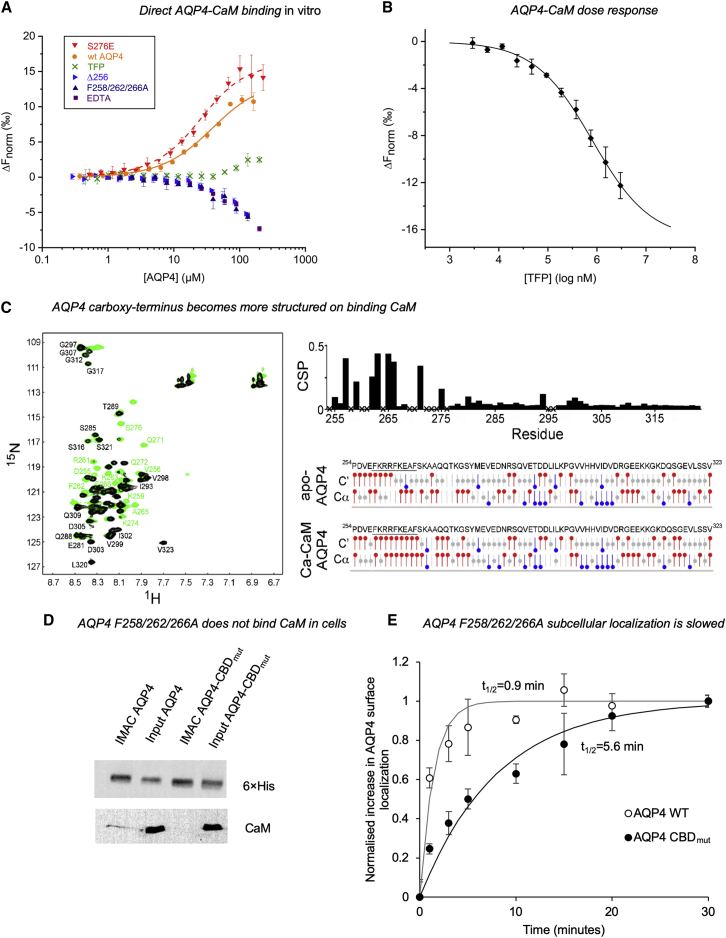
Binding of CaM to the AQP4 Carboxyl Terminus (A) Microscale thermophoresis (MST) data showing that full-length AQP4 interacts directly with CaM. The binding curve, obtained by plotting ΔF_norm_ against [AQP4], could be fitted to a one-to-one binding model (estimated K_d_ of 29 ± 5.6 μM). Addition of 5 mM EDTA demonstrated that binding was Ca^2+^ dependent. The interaction was also inhibited by addition of 1 mM TFP. Truncation of the AQP4 carboxyl terminus before the predicted CBD (AQP4-Δ256) resulted in a construct that did not interact with CaM. AQP4 F258/262/266A did not bind CaM. The phospho-mimetic mutant AQP4-S276E bound CaM with approximately 2-fold higher affinity (K_d_ = 17 ± 3.1 μM) than WT AQP4 (p = 0.031), suggesting that phosphorylation of S276 affects the interaction with CaM. (B) Response curve showing that TFP inhibits the interaction between AQP4 and CaM in a concentration-dependent manner. The concentration of TFP needed for 50% inhibition (IC_50_) was estimated to be 790 ± 2 μM. (C) Left: ^1^H, ^15^N-HSQC NMR data showing the interaction of the recombinant carboxyl terminus of AQP4 (recAQP4ct; residues 254–323) with CaM at 30°C. Chemical shift perturbations (CSPs) were observed by titrating CaM into 0.5 mM ^13^C, ^15^N-labeled recAQP4ct with 0 (black) and 2 (green) molar equivalents of CaM in the presence of 6 mM Ca^2+^. Top right: CSPs induced by CaM binding to recAQP4ct were plotted as a function of the residue number (X indicates that data are not available). Bottom right: chemical shift index (CSI) values of the Cα and C′ atoms of recAQPct. The sequence of the CBD is underlined. The region with consecutive positive CSI values (red) represents an α-helical conformation. (D) Anti-CaM immunoblotting following nickel affinity chromatography (IMAC) from 1% Triton X-100 lysates (input) of HEK293 cells transfected with AQP4-His_6_ (AQP4) or AQP4 F258/262/266A-His_6_ (AQP4 CBD_mut_) demonstrates that the F258A/F262A/F266A mutation abrogates AQP4-CaM binding. (E) Cell-surface biotinylation followed by neutravidin/anti-AQP4 ELISA demonstrates a reduced rate of AQP4 plasma membrane accumulation in AQP4-transfected HEK293 cells upon F258A/F262A/F266A mutation (AQP4 CBD_mut_) compared with the wild-type control (AQP-WT). Normalized data were fitted to functions of the form 1−e^−kt^, and t_1/2_ was calculated as −ln(0.5)/k. See also Figures S4 and S5.

Full-length human AQP4, a carboxy-terminal deletion mutant (AQP4-Δ256), and a triple mutant in which all three phenylalanines were replaced with alanine (AQP4-F258/262/266A) were recombinantly expressed in the yeast *Pichia pastoris*. The ability of all three AQP4 constructs to interact with human CaM was probed using microscale thermophoresis (MST; Figure S5A). The functional integrity of recombinant AQP4 as a water channel was verified using a liposome shrinking assay, from which we calculated an AQP4 single-channel permeability of 1.1 ± 0.1 × 10^−13^ cm^3^/s, in agreement with literature values (Tong et al., 2012, Yang et al., 1997; Figure S5B). As seen in Figure 3A, full-length AQP4 directly interacts with CaM in the presence of Ca^2+^. The binding curve could be fitted to a one-to-one binding model with an estimated K_d_ of 29 ± 6 μM. 5 mM EDTA abolished the interaction, demonstrating that binding was Ca^2+^ dependent. The interaction was also inhibited by 1 mM TFP. AQP4-Δ256 and AQP4-F258/262/266A did not interact with CaM, suggesting that the CBD is located in the carboxyl terminus and involves the predicted hydrophobic surface created by three phenylalanines.

In an additional MST experiment, the AQP4 and CaM concentrations were kept constant, and the concentration of TFP was varied (Figure S5A). The experiment resulted in a concentration-response curve (Figure 3B) showing that TFP inhibits the interaction between AQP4 and CaM in a concentration-dependent manner. The interaction could not be studied at higher TFP concentrations because of significant attenuation of the fluorescence signal from CaM-Alexa Fluor 488. The concentration of TFP needed for 50% inhibition (IC_50_) was 790 ± 2 μM. This is higher than the *in vitro* IC_50_, likely because of partitioning of the hydrophobic TFP molecule into detergent micelles used to solubilize AQP4.

To examine whether the interaction between AQP4 and CaM is affected by AQP4 phosphorylation, S276 of the PKA site was replaced with glutamate (AQP4-S276E), and its interaction with CaM was studied by MST. A binding curve was obtained (Figure 3A), but with approximately 2-fold higher affinity (K_d_ = 17 ± 3 μM) than wild-type AQP4 (p = 0.031), suggesting that the interaction of AQP4 with CaM is strengthened upon phosphorylation.

The structural consequences of the AQP4-CaM interaction were investigated using a ^1^H, ^15^N-HSQC NMR experiment on the recombinant AQP4 carboxyl terminus (recAQP4ct, residues 254–323) with CaM at 30°C. Figure 3C provides direct evidence that CaM interacts with the predicted AQP4 CBD (residues 256–275; Figure S4A) in the presence of Ca^2+^. Chemical shift perturbations induced by CaM binding to recAQP4ct were plotted as a function of the residue number. Chemical shift index values of the Cα and C′ atoms within recAQPct suggested that, upon CaM binding, the AQP4 carboxyl terminus becomes more α-helical in character. CaM binding was accompanied by structural changes that extended beyond the CBD region that harbors the three phenylalanine residues (FKRRFKEAF; Figure 3C), including the region where S276 is located. Further downstream, the carboxyl terminus remained unstructured and was not involved in CaM binding.

### Direct Interaction with CaM Triggers AQP4 Subcellular Relocalization

AQP4 binds CaM *in vitro* (Figure 3A), whereas inhibition with TFP prevented AQP4 relocalization in primary cortical astrocytes (Figure 1A) and CNS edema *in vivo* (Figure 2). To confirm a direct role of AQP4-CaM interaction in AQP4 subcellular relocalization, we studied CaM binding and AQP4 translocation in HEK293 cells transfected with AQP4 wild-type or CBD mutants (F258/262/266A, F258A, F262A, and F266A). HEK293 cells were used rather than astrocytes to avoid mixed endogenous/exogenous AQP4 tetramers; the molecular mechanism of AQP4 translocation is conserved between HEK293 and primary astrocytes (Kitchen et al., 2015). In agreement with our *in vitro* findings (Figure 3A), wild-type AQP4 bound CaM in HEK293 cells whereas AQP4-F258/262/266A did not (Figure 3D). Figure 3E shows a reduced rate of AQP4 plasma membrane accumulation in AQP4-transfected HEK293 cells upon F258/262/266A mutation (AQP4 CBD_mut_; t_1/2_ = 5.6 min) compared with the wild-type control (AQP WT; t_1/2_ = 0.9 min). In contrast, hypotonicity triggered AQP4 relocalization to the plasma membrane for all of the single mutants (F258A, F262A, and F266A) as for WT AQP4 (Figure S4G). These results demonstrate that the AQP4-CaM interaction drives rapid AQP4 subcellular relocalization.

### Attenuation of CNS Edema by Targeted Inhibition of AQP4 Improves Electrophysiological, Sensory, and Locomotor Function

**Figure 4:**
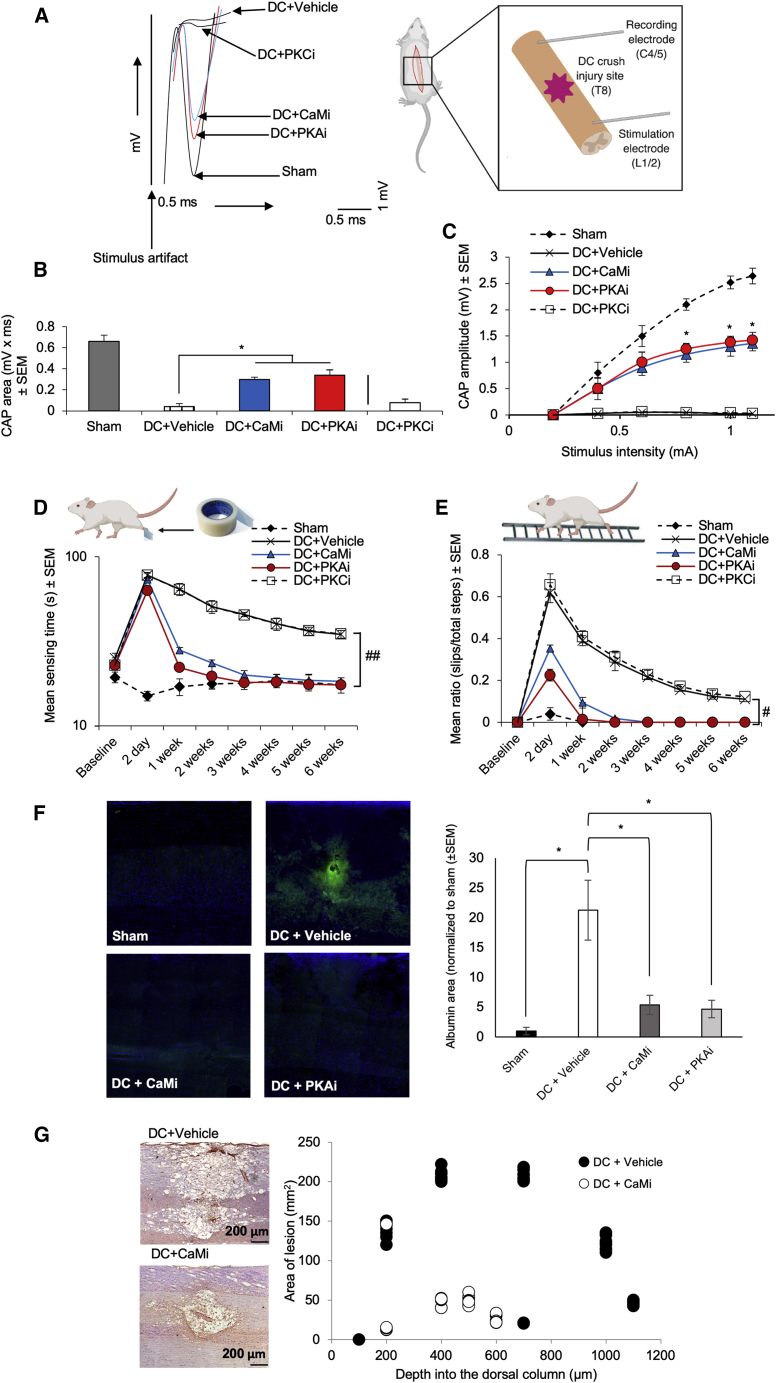
Inhibition of AQP4 Expression and Subcellular Relocalization after Cytotoxic Edema *In Vivo* Improves Electrophysiological, Sensory and Locomotor Functional Recovery, BSCB Breakdown, and Spinal Cord Cavitation (A) Superimposed representative spinal cord compound action potential (CAP) traces demonstrating the significant functional improvement in CaM_i_-treated (TFP) and PKA_i_-treated (H89) rats compared with vehicle-treated and PKC_i_-treated (Gö 6983) rats following DC crush. CaM_i_ treatment was 41 mM TFP. PKA_i_ treatment was 10 mM H89. PKC_i_ treatment was 9.94 μM Gö 6983. In all cases, these were injected directly into the lesion site at a volume of 2.5 μL. (B and C) CaM_i_ and PKA_i_ significantly improved the mean CAP area (B) and the mean CAP amplitude (C). (D) CaM_i_ and PKA_i_ treatment significantly improved the hindpaw tape sensing and removal time, and within 2 weeks, CaM_i_-treated and PKA_i_-treated rats recovered completely and were indistinguishable from sham controls. (E) The mean ratio of slips/total steps in a ladder-crossing test was significantly improved after CaM_i_ and PKA_i_ treatment, returning to sham control levels by 2 weeks. For the tape sensing/removal and ladder crossing tests, significant deficits remained in vehicle-treated and PKC_i_-treated rats. #p < 0.05, linear mixed model; ##p < 0.05, generalized linear mixed model; calculated as described previously (Fagoe et al., 2016; Table S2; n = 6 rats/group; 3 independent repeats; total n = 18 rats/group). (F) Treatment with 41 mM TFP significantly reduced BSCB breakdown 7 days after DC crush (right panel), as determined by albumin staining (green) around the lesion site (representative images are shown in the left panel). ^∗^p < 0.05 (Table S2) by ANOVA followed by t test with Bonferroni correction. (G) TFP also suppressed the normal process of cavitation that occurs at lesion sites after DC crush, significantly reducing the cavity area 6 weeks after DC crush at all depths through the DC (right panel; black-filled dots represent DC+vehicle; white dots represent DC+CaM_i_), as determined by laminin staining (brown) (representative images are shown in the left panel). n = 6 rats/group; 2 independent repeats (total n = 12 rats/group); p = 4.0 × 10^−10^, comparing lesion volume in DC+vehicle with DC+CaM_i_ by t test (Table S2). See also Figure S5 and Table S1.

Compound action potentials (CAPs) were recorded across the lesion site as a measure of neuronal signal conduction following DC crush injury with or without treatment (Figures 4A and S5C; Table S1). The negative (or repolarization) CAP trace across the DC lesion site (at T8) was ablated after DC crush injury (Almutiri et al., 2018, Hains et al., 2004, Lo et al., 2003) but was significantly restored following treatment with the CaM inhibitor TFP or the PKA inhibitor H89 (Figure 4A). The PKC inhibitor Gö 6983 had no effect on the ablated negative CAP trace, which was similar to that of injured, untreated rats (DC+vehicle; Figure 4A). The CAP area (Figure 4B) and the mean CAP amplitude (Figure 4C) were also significantly improved following treatment with TFP or H89 but not with Gö 6983. The mean CAP areas 6 weeks after injury in DC+CaM_i_- and DC+PKA_i_-treated rats were 45.5% ± 0.2% and 52.5% ± 0.7% of that observed for control animals (sham), respectively. Overall, treatment with TFP or H89 reduced injury-induced deficits.

We determined whether these electrophysiological improvements translated into improvements in sensory and locomotor function in treated rats. To assess sensory recovery, we used the tape sensing and removal test, in which a small piece of sticky tape is adhered to the hind paw; the time taken for rats to detect and remove the tape is recorded (Almutiri et al., 2018; Figure 4D). To assess locomotor function, we used the horizontal ladder crossing test, in which rats navigate a 0.9-m ladder with rungs randomly spaced 3.5–5.0 cm apart and the number of slips/footfalls versus the total number of steps taken to cross the ladder is recorded (Almutiri et al., 2018; Figure 4E). These tests are sensitive in discriminating subtle changes in sensory and locomotor function after DC crush injury (Almutiri et al., 2018, Fagoe et al., 2016).

The mean sensing and removal time in injured (DC+vehicle) animals increased from 25 s at baseline to 78 s at 2 dpi (Figure 4D). In sham animals, the mean sensing and removal time was 15–20 s throughout the 6-week assessment period, whereas after injury (DC+vehicle), a slight improvement was observed throughout the time period. However, significant deficits remained at 6 weeks, with animals taking an average of 35 s to sense and remove the tape (Figure 4D). In rats treated with TFP (DC+CaM_i_) or H89 (DC+PKA_i_), significantly reduced sensing and removal times were observed within 1 week after injury, and these rats were indistinguishable from control animals (sham) 3 weeks post-injury (Figure 4D). No improvements in tape sensing and removal times were observed in animals receiving the PKC inhibitor Gö 6983 (DC+PKC inhibition [PKC_i_]); responses were similar to injured, untreated rats (DC+vehicle). Similarly, there was a significant deficit in ladder-crossing performance in injured, untreated rats (DC+vehicle) and rats treated with the PKC inhibitor Gö 6983, which remained throughout the 6-week assessment period (Figure 4E). In rats treated with TFP or H89 (DC+CaM_i_ or DC+PKA_i_), significant improvements in ladder-crossing performance were observed at 2 dpi, with animals being indistinguishable from control (sham) animals by 2 weeks after injury. Limiting DC crush injury-induced CNS edema with TFP or H89 restored sensory and locomotor function in adult rats, with their ability to walk returning to pre-injury levels.

### TFP Reduces BSCB Breakdown and Cavity Size 6 Weeks after Injury

We evaluated whether TFP treatment affected BSCB breakdown and lesion cavity size, which occur in humans and adult rats after DC crush injury (Surey et al., 2014). BSCB integrity is commonly assessed by immunohistochemically detecting albumin extravasation into the spinal cord parenchyma after injury (Cohen et al., 2009). Albumin immunohistochemistry and subsequent quantification showed that the large area of BSCB breakdown in injured, untreated animals (DC+vehicle) was significantly reduced (4.0-fold) in TFP-treated rats (DC+CaM_i_; Figure 4F). This was accompanied by a 4.0-fold reduction in lesion cavity size (p = 4.0 × 10^−10^ versus DC+vehicle) at all depths through the lesion site (Figure 4G). These results demonstrate that TFP suppresses BSCB breakdown and reduces lesion cavity size, both of which are likely to contribute to the improvements in sensory and locomotor function in TFP-treated rats.

## Discussion

CNS edema is caused by traumatic injuries, infection, tumor growth, and stroke (Jha et al., 2019, Liang et al., 2007). Traumatic injuries are a leading cause of psychiatric disorders, substance abuse, attempted suicide, disability, and early death in adults under 45 years of age (Fazel et al., 2014). The biggest increase in patient numbers is currently in those older than 60 years (so-called “silver trauma”). In the United States, this silent epidemic affects more than 1.7 million individuals annually. Depression, suicidal behavior, and an increased risk of neurodegenerative conditions such as Alzheimer’s disease and Parkinson’s disease are known outcomes of traumatic CNS injury in patients of any age (Chen et al., 2014, Zlokovic, 2011).

Although industry has pursued the development of specific drugs that halt CNS edema progression, all have failed in phase III clinical trials; two recent trials showed that progesterone had no effect on neurological outcome following TBI (Stein, 2015). Notably, few strategies have focused on the primary cause of CNS edema, which is dysregulated flow of water into cells. Current treatment approaches are therefore limited by an absence of pharmacological interventions and a reliance on alleviating the symptoms of edema and not the causes, using therapies introduced more than 80 years ago (Manley et al., 2000).

AQPs play an essential role in promoting short-term susceptibility to the pathological changes in volume that enhance CNS edema formation; consequently, they are established as drug targets (Verkman et al., 2014). All previous strategies to identify AQP inhibitors have focused on blocking the pore of the AQP channel. Based on this approach, pharmacological inhibition of AQP4 by AER-270 has been suggested to cause a reduction in CNS edema, and AER-271, a pro-drug of AER-270, is the subject of phase I safety trial NCT03804476 in healthy volunteers. However, although 70% maximal inhibition of rat AQP4 and 20% maximal inhibition of mouse and human AQP4 have been reported in water transport assays, AER-270 had the same effect on water content in rat and mouse stroke models, suggesting that the effect is not AQP4 dependent (Farr et al., 2019). Because AER-270 is a known nuclear factor κB (NF-κΒ) inhibitor, and NF-κΒ inhibition can reduce CNS water content (Li et al., 2016), it may instead be acting through this modality.

We show a direct mechanistic relationship between inhibition of AQP4 function and a reduction in CNS edema. We present an *in vivo* demonstration that targeting the subcellular localization of a membrane channel protein, rather than targeting its activity directly, is a viable therapeutic strategy. Our focus on targeting a fundamental cellular process, rather than trying to block a pore, provides a broadly applicable framework for future drug development. Regulation by vesicular trafficking is a common biological mechanism controlling the function of many membrane protein families (Offringa and Huang, 2013). It is well-characterized for AQP2 in the renal collecting duct in response to the antidiuretic hormone vasopressin (van Balkom et al., 2002), but current dogma fails to recognize the central role of translocation as a regulatory mechanism for the AQP family as a whole (especially in response to non-hormonal, physiological triggers) and its implications for cell and tissue homeostasis. Here we present pathophysiologically relevant AQP subcellular relocalization and establish it as a regulatory mechanism. Using a rat model of CNS edema, we show that CaM_i_ or PKA_i_ effectively limits spinal cord water influx 3 dpi and completely abolishes spinal cord edema by 7 dpi (Figure 2; Figure S2 shows the same effect after brain edema). Total AQP4 expression and localization at the BSCB increased following DC crush injury, and both were blocked by inhibition of CaM or PKA (Figure 2). We also demonstrate the central roles of CaM and PKA in increased cell-surface expression of AQP4 and AQP4 subcellular relocalization to the BSCB following injury (Figures 1 and 2). Ablation of edema and AQP4 BSCB relocalization *in vivo* are accompanied by complete functional recovery by 2 weeks post-injury (Figure 4). Although CaM has multiple roles in our proposed mechanism, we demonstrate a direct interaction between CaM and AQP4 that is necessary for AQP4 subcellular relocalization (Figure 3).

The anti-psychotic effects of TFP are attributed to its anti-dopaminergic and anti-adrenergic actions (Qin et al., 2009). Dopamine decreases AQP4 expression in cultured astrocytes (Küppers et al., 2008); if the anti-dopaminergic effects of TFP were dominant in our experiments, then we would expect increased AQP4 expression following TFP treatment and worsening of edema, as seen when the selective D2 antagonist L-741,626 increased spinal cord water content above the DC+vehicle control 3 dpi (Figure 2B). The fact that we observed the opposite with TFP suggests that the CaM_i_ effects of TFP dominate any anti-dopaminergic effects in CNS edema. This increase in edema with an anti-dopaminergic inhibitor along with the increased edema following PKC_i_ is particularly interesting, given that, in the cultured pig kidney cell line LLC-PK1, dopamine signaling via PKC reduced AQP4-dependent plasma membrane water permeability (Zelenina et al., 2002); our data suggest that this pathway may also be active in astrocytes. Anti-adrenergics are associated with delayed onset of edema after intracerebral hemorrhage in humans but have no effect on patient outcome (Sansing et al., 2011). We see prevention of edema following TFP treatment (rather than just delayed onset) and a strong effect on outcome, whereas the α1 antagonist terazosin had no effect on spinal cord water content following DC crush injury. This suggests that the primary modality of TFP in our model is its CaM antagonism (Tanokura and Yamada, 1986). Previously published data using a rat model of stroke demonstrate that treatment with TFP prevents onset of brain edema, which has been proposed to be via CaM_i_ stabilizing the integrity of the BBB. We suggest that the beneficial effects are due to a reduction in AQP4 peri-endothelial localization (Figure 2), which was not measured in that study (Sato et al., 2003).

**Figure 5:**
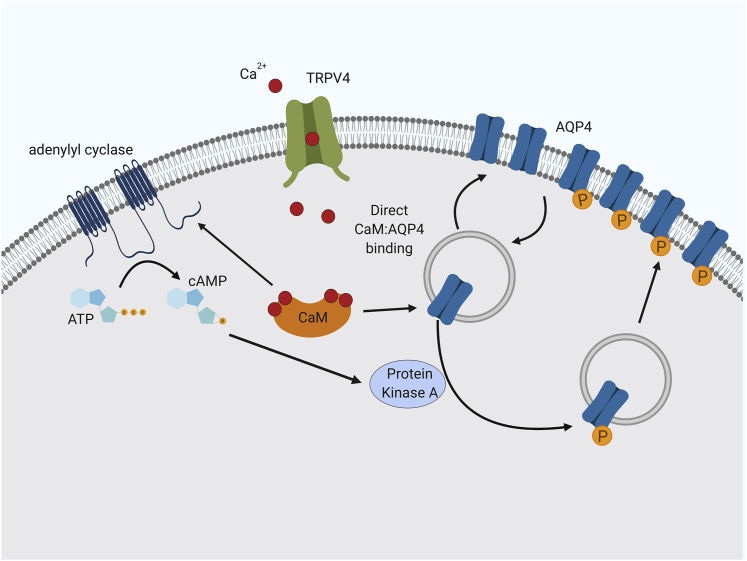
AQP4 Subcellular Relocalization Drives Cytotoxic Edema: The Proposed Roles of CaM and PKA For a Figure360 author presentation of this figure, see https://doi.org/10.1016/j.cell.2020.03.037. Following hypoxic insult, failure in Na^+^, K^+^, and Cl^−^ pumps in the plasma membrane leads to osmotic dysregulation. The mechanosensitive TRPV4 channel facilitates an influx of Ca^2+^ ions into astrocytes, which activates CaM. CaM interacts with an adenylyl cyclase, activating cyclic AMP (cAMP)-dependent PKA, which phosphorylates AQP4 at Ser276, causing it to relocalize to the plasma membrane. CaM interacts directly with AQP4; this regulatory interaction drives AQP4 subcellular relocalization (created with https://biorender.com).

Our data show that CaM has at least two distinct roles in translocation of AQP4 in astrocytes. First, activation of CaM following opening of TRPV4 (which has been suggested to interact with AQP4; Benfenati et al., 2011, Jo et al., 2015) leads to activation of an adenylyl cyclase, production of cAMP, and activation of PKA (Figure 1). Second, CaM binds directly to AQP4 (Figure 3), and the strength of this binding is modulated by phosphorylation of AQP4 at a single PKA consensus site, S276. We have shown previously (Kitchen et al., 2015) that hypotonicity-mediated AQP4 relocalization is blocked by several PKA inhibitors (hypericin, H-89, and myr-PKI, in increasing order of specificity), that a non-phosphorylatable AQP4 mutant (S276A) does not relocalize, and that a phospho-mimetic mutation (S276D) removes the PKA dependence of the relocalization. Furthermore, multiple phosphoproteomics datasets (retrieved via dbPAF; Ullah et al., 2016; available at http://dbpaf.biocuckoo.org) demonstrate that AQP4-S276 is phosphorylated in human, rat, and mouse tissue samples. Together, these data suggest a model of AQP4 translocation whereby an influx of calcium ions activates CaM; this activates PKA via a CaM-activated adenylyl cyclase (e.g., AC1, AC3, or AC8; all three are expressed in rat and mouse brain; Sanabra and Mengod, 2011). PKA phosphorylates AQP4, and CaM binds to AQP4 to facilitate its translocation to the plasma membrane (Figure 5). Our NMR data suggest that this binding causes a conformational change in the AQP4 carboxyl terminus, which becomes more structured, with increased α-helical content (Figure 3C). An increased affinity toward the AQP4-S276E phospho-mimetic mutant suggests that CaM preferentially binds phosphorylated AQP4, with a 2-fold decrease in K_d_. Although this increase in affinity is modest, it is possible that the additional charge found on an actual phosphoserine residue compared with the mimetic glutamate residue leads to a more enhanced difference *in vivo*.

CaM and PKA also appear to be involved in the post-injury increase in AQP4 protein expression. A previous study using a mouse TBI model found that direct activation of the *Aqp4* gene by the transcription factor Foxo3a is responsible for increased AQP4 expression (Kapoor et al., 2013); our data suggest that this response is conserved in rats (Figures 2 and S3). We demonstrate that astrocyte swelling initiates Foxo3a nuclear translocation via PKA-mediated inactivation of Akt. The Akt agonist SC79 has been shown recently to have a neuroprotective effect in a rat middle cerebral artery occlusion (MCAO) model of stroke (Luan et al., 2018). This protective effect was attributed to inhibition of apoptosis; our data suggest that an alternative interpretation of these findings is inhibition of *Aqp4* upregulation in astrocytes following MCAO. Previous studies showing increases in AQP4 surface localization used a model of astrocytic cell swelling based on hypotonic treatment of astrocytes (Kitchen et al., 2015, Salman et al., 2017a). Although this creates a tonicity gradient similar to that seen during cytotoxic edema formation after stroke or traumatic injury, physiologically, astrocytes actually experience an intracellular increase in tonicity relative to the extracellular fluid caused by hypoxia-driven effects on ion channels and transporters (Lafrenaye and Simard, 2019). Our data in Figures 1A–1D provide evidence of acute hypoxia-mediated AQP4 translocation and are the basis of a simple and easy-to-implement *in vitro* model to study CNS injury and edema, which reproduces the AQP4 translocation response observed *in vivo*.

Following injury, increased AQP4 immunoreactivity (Figure 2H) might be influenced by astrogliosis. Hypertrophy and migration of reactive astrocytes (which have increased AQP4 expression; Vizuete et al., 1999) into the injury epicenter aid tissue repair and cause glial scarring. Astrocyte migration depends on AQP4 at the leading edge (Saadoun et al., 2005); inhibiting AQP4 relocalization may reduce the number of infiltrating reactive astrocytes. An additional benefit of inhibiting AQP4 relocalization may therefore be a reduction in the number of invading, reactive astrocytes and in the extent of glial scarring. This may facilitate axon sprouting and could account for the improved electrophysiological outcomes reported in our study.

TFP is licensed as a drug for human use (NICE, 2019) that we administered in rats at a dose approximately equivalent to its licensed dose in humans. This treatment resulted in functional recovery 2 weeks after DC crush injury; animals treated with TFP could walk normally after 2 weeks, whereas untreated animals had still not recovered 6 weeks post-injury. The future socio-economic impact of this work is enormous; our data provide a molecular mechanistic understanding of water channel regulation (Figure 5) that has the potential to define a therapeutic framework for the tens of millions of CNS edema patients annually, worldwide, for whom there is still no pharmacological intervention.

## STAR★Methods

### Key Resources Table

**Table:** 

REAGENT or RESOURCE	SOURCE	IDENTIFIER
**Antibodies**		

Rabbit polyclonal anti-AQP4 antibody	Abcam	Cat#:ab46182; RRID: AB_955676
Mouse monoclonal anti-AQP4 antibody	Abcam	Cat#:ab9512; RRID: AB_307299
Rabbit anti-AQP4 antibody	Abcam	Cat#:ab128906; RRID: AB_11143780
Chicken anti-mouse IgG-HRP antibody	Santa Cruz	Cat#:sc-2954; RRID: AB_639239
Donkey anti-rabbit IgG-HRP antibody	Santa Cruz	Cat#:sc-2313; RRID: AB_641181
Rabbit anti-Foxo3 IgG antibody	Abcam	Cat#:ab23683; RRID: AB_732424
Mouse anti-RECA-1 antibody	Abcam	Cat#:ab9774; RRID: AB_296613
Goat anti-rabbit IgG FITC antibody	Merck	Cat#:F0382; RRID: AB_259384
Goat anti-chicken IgY Alexafluor 488 antibody	Abcam	Cat#:ab150169; RRID: AB_2636803
Goat anti-mouse IgG Alexafluor 633 antibody	ThermoFisher	Cat#:A-21050; RRID: AB_141431
Chicken anti-albumin antibody	Abcam	Cat#:ab106582; RRID: AB_10888110
Rabbit polyclonal anti-laminin antibody	Sigma	Cat#:L9393; RRID: AB_477163
Mouse anti β-actin antibody	Sigma-Aldrich	Cat#:A2228; RRID: AB_476697
Rabbit anti-calmodulin antibody	Cell Signaling	Cat#:D1F7J; RRID: AB_2799090
Rabbit anti-lamin B1 antibody	Cell Signaling	Cat#:D4Q4Z; RRID: AB_2650517
Rabbit anti-alpha tubulin antibody	Cell Signaling	Cat#:2144; RRID: AB_2210548
Mouse anti-His tag antibody	Takara Bio	Cat#:631212; RRID: AB_2721905

**Bacterial and Virus Strains**		

*E.coli* strain DH5_α_	Thermo Fisher	18265017
One Shot BL21 (DE3) *E. coli*	Thermofisher	C600003
One Shot TOP10 *E. coli*	Thermofisher	C404010

**Chemicals, Peptides, and Recombinant Proteins**		

Trifluoperazine dihydrochloride	Sigma-Aldrich	T8516
W-7 hydrochloride	Sigma-Aldrich	681629
L-741,626	Sigma-Aldrich	L135
Terazosin hydrochloride	Sigma-Aldrich	T4680
H89	Tocris	2910
Gö6983	Tocris	2285/1
1-Palmitoyl-2-oleoyl-sn-glycero-3-phosphocholine (POPC)	Sigma	42773
2-Oleoyl-1-palmitoyl-sn-glycero-3-phospho-rac-(1-glycerol) (POPG)	Sigma	76559
Cholesterol	Sigma	C8667
5(6)-Carboxyfluorescein	Sigma	21877
Alexa Fluor 488 maleimide	ThermoFisher	A10254
NH_4_Cl (^15^N, 99%)	Cambridge Isotopes	NLM-467-5; CAS# 39466-62-1
D-glucose (1-13C, 98-99%)	Cambridge Isotopes	CLM-1396-5; CAS# 110187-42-3
Glutathione Sepharose 4B	GE Healthcare	17-075601
PreScission protease	GE Healthcare	27-084301
Phos-Tag acrylamide	NARD Institute	AAL-107
2,2-dimethyl-2-silapentane-5-sulfonate	Cambridge Isotopes	DLM-32-10; CAS# 2039-96-5
D_2_O (D, 99.96%)	Cambridge Isotopes	DLM-6-1000; CAS# 7789-20-0
Amicon Ultra-15 centrifugal filter unit	Millipore-Sigma	UFC900308
M9 minimal media salts	Millipore-Sigma	M9956-500ML
Recombinant human AQP4 (UniProtID P55087)	Öberg et al., 2011	N/A
Recombinant human AQP4- Δ256	This paper	N/A
Recombinant human AQP4-F258/262/268A	This paper	N/A
Recombinant human AQP4-S276E	This paper	N/A
Recombinant GST-recAQP4ct (containing human AQP4 residues 254-323)	This paper	N/A
Recombinant human calmodulin S17C (UniProtID P0DP23)	O’Connell et al., 2010	N/A
Recombinant chicken calmodulin	Nakashima et al., 1996	N/A
Bovine heart cAMP-dependent protein kinase (PKAc)	Hofmann et al., 1975	N/A
EZ-Link Sulfo-NHS-SS-biotin, cell impermeable biotinylation reagent	ThermoFisher	21331
Calcein-AM	ThermoFisher	C3100MP

**Critical Commercial Assays**		

RNeasy Plus Mini Kit	QIAGEN	74134
QIAquick PCR Purification Kit	QIAGEN	28104
Pierce BCA Protein Assay Kit	ThermoFisher	23225
ELISA-based assay kit for PKA activity	Abcam	ab139435
ELISA-based assay kit for Akt activity	Abcam	ab139436

**Experimental Models: Cell Lines**		

Rat primary cortical astrocytes	GIBCO	N7745100
Human cerebral cortex primary astrocytes	Sciencell	1800
HEK293	ATCC	CRL-1573

**Experimental Models: Organisms/Strains**		

Rats/Sprague-Dawley	Charles River UK	CD IGS
*Pichia pastoris* strain X-33	Thermo Fisher	C18000

**Oligonucleotides**		

Primer recAQP4ct: NT forward 5′-GCGCGGATCCCCAGATGT TGAATTCAAACG	This study	N/A
Primer recAQP4ct: NT reverse 5′-CCATCTGGAGAGGTATT GTCTTCA	This study	N/A
Primer recAQP4ct: CT forward 5′-CAATCTGGAGAGGTATT GTCTTCAGTATAAGCGGCCGCGCGC	This study	N/A
Primer recAQP4ct: CT reverse 5′- GCGCGCGGCCGCTTAT ACTGAAGACAATACCTCTCCAGATTG	This study	N/A

**Recombinant DNA**		

pGFP-C-shLenti-AQP4	Origene	Cat no. TL709442, Locus ID: 25293
pGFP-C-shLenti-Control	Origene	Cat no. TR30021
pDEST47-hAQP4-His_6_	This study	N/A
pDEST47-hAQP4-F258A-His_6_	This study	N/A
pDEST47-hAQP4-F262A-His_6_	This study	N/A
pDEST47-hAQP4-F266A-His_6_	This study	N/A
pDEST47-hAQP4-F258/262/266A-His_6_	This study	N/A
pGEX-6P1	GE Healthcare	27-1542-01

**Software and Algorithms**		

NMRPipe	NIST IBBR	https://www.ibbr.umd.edu/nmrpipe/
NMRViewJ	NMRFx	http://nmrfx.org/nmrfx/nmrviewj

### Lead Contact and Materials Availability

Further information and requests for resources and reagents should be directed to and will be fulfilled by the Lead Contact, Roslyn M. Bill (r.m.bill@aston.ac.uk). All unique/stable reagents generated in this study are available from the Lead Contact with a completed Materials Transfer Agreement.

### Experimental Model and Subject Details

#### Cell culture

Rat primary cortical astrocytes (Invitrogen, Poole, UK) were cultured routinely in DMEM supplemented with 20% (v/v) fetal bovine serum (Invitrogen), 1% penicillin/streptomycin and 1% glutamine in humidified 5% (v/v) CO_2_ in air at 37°C. Primary human cortical astrocytes (Sciencell, Cat. No. 1800) were plated on 75 cm^2^ culture flasks (Thermo Scientific Nunc Cell Culture Treated EasYFlasks) and cultured routinely in Astrocyte Medium (Sciencell; 1801) containing 1% fetal bovine serum (FBS, Sciencell Cat. No. 0010), 5 mL astrocyte growth supplement (AGS, Sciencell Cat. No. 1852) and 5 mL penicillin/streptomycin solution (P/S, Sciencell Cat. No. 0503). The cells were then incubated either in humidified 5% (v/v) CO_2_ in air at 37°C for the normoxia work or in a controlled hypoxic atmosphere using a Coylab Hypoxia Chamber Glove Box, with a humidified airtight apparatus with inflow and outflow valves (into which a mixture of 90% N_2_, 5% O_2_ and 5% CO_2_ was flushed) for the hypoxia-related experiments. HEK293 cells were cultured routinely in DMEM supplemented with 10% (v/v) fetal bovine serum (Invitrogen). HEK293 cells were transiently transfected when approximately 70% confluent, using 12 μg polyethyleneimine (branched, average M_r_ = 25,000, Sigma-Aldrich, 408727) and 2 μg plasmid DNA in 35 mm dishes.

#### DC crush injury in adult rats

All animal experiments were licensed by the UK Home Office and experimental protocols approved by the University of Birmingham’s Animal Welfare and Ethical Review Board. All animal surgeries were carried out in strict accordance with the guidelines of the UK Animals Scientific Procedures Act, 1986 and the Revised European Directive 1010/63/EU and conformed to the guidelines and recommendation of the use of animals by the Federation of the European Laboratory Animal Science Associations (FELASA). The ARRIVE guidelines for reporting of *in vivo* experiments were followed. Adult, female Sprague-Dawley rats weighing 170-220 g (Charles River, Margate, UK) were randomly assigned to each experimental group with the investigators masked to the treatment conditions. The rats used were 6-8 weeks old at the start of each experiment. Rats were injected subcutaneously with 50 μl buprenorphine to provide analgesia prior to surgery and anaesthetized using 5% isoflurane in 1.8 ml/l of O_2_ with body temperature and heart rate monitored throughout surgery. To injure the DC axons, a partial T8 laminectomy was performed: the DC was crushed bilaterally using calibrated watchmaker’s forceps (Lagord et al., 2002, Surey et al., 2014). Experiments investigating the effects of inhibitors of AQP4 relocalization (or control inhibitors) on CNS edema comprised the following groups: Sham controls (partial laminectomy but no DC lesion); DC+Vehicle (partial laminectomy followed by T8 DC crush + intra-lesional injection of vehicle (PBS); DC+CaM_i_ (partial laminectomy followed by T8 DC crush + intra-lesional injection of 5 μg TFP in 2.5 μl final volume; 41 mM); DC+PKAi (partial laminectomy followed by T8 DC crush + intra-lesional injection of 10 μM H89 (PKAi)); DC+PKCi (partial laminectomy followed by T8 DC crush + intra-lesional injection of 9.94 μM Gö 6983 (PKCi)). Additional inhibitors were CaM_i_ (164 mM W-7), A1R_i_ (53 mM terazosin) and D2R_i_ (6.6 mM L-741,626). All inhibitors were injected in a final volume of 2.5 μl. Intra-lesional injections were performed using in-house pulled glass micropipettes (P1000 micropipette puller set to pull micropipettes with an internal tip diameter of 0.5 μm; Sutter Instruments, Novato, CA, USA). The glass micropipettes were loaded with appropriate solutions, attached to the rubber tubing from butterfly needles and secured to a 5 mL syringe. Using a dissecting microscope, the tip of the micropipette was inserted into the lesion site and solutions were pushed through, with air in the syringe, slowly over a 1 minute period, with a 20 s wait prior to withdrawal of the micropipette to alleviate backflow. To assess the effect of short hairpin RNA to AQP4 (shAQP4) on edema and behavioral recovery after DC crush injury, groups comprised DC+shControl (scrambled control driven by a U6 promoter; Cat No. TR30021, Origene, Rockville, MD, USA) and DC+shAQP4 (U6 promoter + shAQP4; Cat No. TL709442, Origene) were investigated. 2 μg plasmid DNA was injected directly into the dorsal root ganglion (DRG) using micropipettes as described above, immediately after DC crush injury (Almutiri et al., 2018). *In vivo* jetPEI (PEI: Polyplus Transfection, New York, USA) was prepared according to the manufacturer’s instructions and 2 μg plasmid DNA was used to transduce DRG neurons (DGRN). Non-viral DRGN transduction is as effective as AAV but without the need to transduce DRGN 1-2 weeks prior injury to maximize transgene expression (Almutiri et al., 2018). All rats were housed under standard conditions after surgery along with their cage mates in groups of 4. Animals were allowed to survive for either 3-28 days for water content analysis or 6 weeks for electrophysiological, behavioral and histological assessments.

#### Cortical stab injury model in adult rats

A modified 3 mm cortical stab injury, originally described in the mouse, was adapted for adult Sprague-Dawley rats (Allahyari and Garcia, 2015). Adult, female Sprague-Dawley rats weighing 170-220 g (Charles River, Margate, UK) were randomly assigned to each experimental group with the investigators masked to the treatment conditions. Briefly, after securing the rat’s head in a stereotaxic frame, a small craniotomy was performed. A number 11 blade was then attached to the manipulator arm of the stereotaxic frame and lowered precisely 3 mm into the brain tissue, 1 mm caudal to the coronal suture and 1 mm lateral to the sagittal suture and kept in place for 10 s prior to removal. A small piece of gel foam was then used to soak up any excess blood and fluids. Animals were treated with intra-lesional injections with pulled glass micropipettes with CaM_i_ (Stab injury+CaMi), PKA_i_ (Stab injury+PKA_i_) and PKC_i_ (Stab injury+PKC_i_) or Vehicle (Stab injury). Untreated (Sham) animals had a craniotomy only. The skull flap was replaced and kept in place with bone cement. The skin was then closed using absorbable sutures and animals returned to their home cages.

### Method Details

#### ELISA

AQP4 protein levels were measured by sandwich ELISA following the manufacturer’s instructions (Abcam) (Salman et al., 2017b). A 96-well plate (Nunc, Wiesbaden, Germany) was coated by overnight incubation at 4°C with 5 μL/well of rabbit polyclonal anti-AQP4 (Abcam, ab46182) diluted 1:500 in carbonate/bicarbonate buffer (pH 9.6). The plate was washed twice with phosphate-buffered saline and 0.05% Tween 20 (PBS-T; pH 7.5). The remaining unsaturated protein-binding sites were blocked with 5% non-fat dry milk in PBS overnight at 4°C with gentle shaking. Plates were washed twice for 5 min with PBS-T. Proteins were extracted using CelLytic™ (Sigma, Cat. No. C2978) supplemented with protease inhibitor cocktail (Sigma, Cat. No. P2714, 1:100). Total protein concentration was determined using Pierce™ BCA Protein Assay Kit (ThermoFisher Scientific, Cat. No. 23225) following the manufacturer’s procedure. 60 μg total protein was added to each well and incubated for 120 min at 37°C. Plates were washed twice with PBS-T. 100 μl of 1:1000 diluted mouse monoclonal anti-AQP4 antibody (Abcam, ab9512) was added to each well. The plates were covered with adhesive plastic and incubated for 2 h at 37°C and then washed twice for 5 min with PBS-T. 100 μL of horseradish peroxidase (HRP)-conjugated secondary antibody, chicken anti-mouse (Santa Cruz, sc-2954), diluted 1:5000 in blocking buffer, was added to each well and incubated for 30 min at 37°C. The plates were washed four times with PBS-T, followed by a single wash with PBS. The plates were incubated with 100 μl /well of RayBio™ TMB One-Step Substrate Reagent (Raybiotech; Cat. No. J120215098), at room temperature for 30 minutes, under light-protected conditions. After the color was developed, the reaction was stopped by adding 50 μl of 2 M H_2_SO_4_. Absorbance was measured at 450 nm using a Perkin Elmer Wallac 1420 Victor2 microplate reader.

#### Cell surface biotinylation

Primary human astrocytes were plated in 6 well plates 2 days before each experiment. Cell surface amines were biotinylated using a cell impermeable amine-reactive biotinylation reagent (EZ-Link Sulfo-NHS-SS-Biotin; ThermoFisher Scientific, Loughborough, UK, Cat. No. 21331). Cells were exposed to the indicated experimental conditions and then incubated in 1 mL of 0.5 mg/ml biotinylation reagent in PBS on ice for 30 minutes. Unreacted reagent was quenched in 500 μL 25 mM glycine in PBS per well for 3 × 5 minutes. Cells were lysed in 250 μl of CelLytic™ (Sigma, Poole, UK, Cat. No. C2978) supplemented with protease inhibitor cocktail (Sigma, Cat. No. P2714, 1:100). The lysate was centrifuged at 21,000 g at 4°C for 10 minutes to remove insoluble material. Biotinylated proteins were pulled out by incubation in Pierce NeutrAvidin Coated Plates, 96-well (ThermoFisher Scientific; Cat. No. 15129) for 2 hours at 4°C with shaking. Each lysate was loaded in triplicate with the same amount of total cellular protein per lysate measured by Pierce BCA Protein Assay Kit (ThermoFisher Scientific, Cat. No. 23225). Plates were blocked with 3% w/v BSA in PBS for 1 hour at RT with shaking. Plates were incubated on a shaker overnight at 4°C with an anti-AQP4 antibody (Abcam, Cambridge, UK, Cat No. ab128906) diluted 1:500 in 0.05% PBS-Tween. Plates were washed with 0.1% PBS-Tween and incubated at RT for 1 hour with HRP-conjugated secondary antibody (Santa Cruz, CA, USA, Cat No. sc-2313) diluted 1:2,500 in 0.05% PBS-Tween. Plates were washed with 0.1% PBS-Tween three times then once with PBS and incubated with SIGMAFAST OPD (Sigma; Cat No. P9187) for 30 minutes, wrapped in foil. Absorbance was measured at 450 nm using Perkin Elmer Wallac 1420 Victor2 microplate reader.

#### Calcein fluorescence quenching

Cells were plated into black-walled, clear-bottomed tissue culture treated 96-well plates (Greiner) 48 hours before the experiment. Cells were loaded with 5 μM calcein-AM in growth medium supplemented with 1 mM probenecid (to inhibit dye leakage) for 90 minutes. Cells were washed twice with HEPES-buffered growth medium supplemented with 1 mM probenecid, then covered with 75 μL probenecid-supplemented HEPES-buffered medium. Fluorescence was read on a BioTek synergy HT plate reader with injector system. Each well was read continuously (dt = 50 ms) for 5 s, followed by injection of 75 μL HEPES-buffered medium containing 400 mM mannitol to give a final concentration of 200 mM and an osmotic gradient of 200 mOsm. Fluorescence was read for a further 50 s. Normalized fluorescence values were converted to normalized volumes using a Coulter counter generated standard curve. Single-phase exponential decay functions were fitted and rate constants were taken as proportional to the membrane water permeability.

#### Analysis of kinase activity

ELISA-based assay kits (Abcam, Cat No. ab139435, ab139436) were used to measure PKA and Akt activity in cell lysates according to the manufacturer’s instructions. Cells were lysed in a non-denaturing, phosphatase inhibiting lysis buffer for 45 minutes, centrifuged at 21,000 g for 10 minutes to remove insoluble material and protein was quantified using detergent-insensitive Bradford assay (Expedeon, San Diego, CA, USA). Lysates were diluted in the kit dilution buffer as required to load 2,000 ng of total cellular protein in 30 μL per well of the assay plate. For *in vitro* phosphorylation, protein kinase A was purified from bovine heart (Hofmann et al., 1975) using DEAE-cellulose chromatography, ammonium sulfate precipitation and size-exclusion chromatography.

#### Expression and purification of recombinant human AQP4 constructs

Full-length AQP4 containing a carboxy-terminal His_6_-tag was expressed in *Pichia pastoris* (Öberg et al., 2011). Constructs containing the phospho-mimetic mutation (AQP4-S276E), a stop codon after residue 256 (AQP4-Δ256) and the phenylalanine triple mutant (AQP4-F258/262/266A) were generated using a megaprimer mutagenesis protocol (Tseng et al., 2008). Cells were grown in a 3 L fermentor (Belach Bioteknik, Stockholm, Sweden) and protein expression was induced using methanol for 24-36 hours. Routinely, 50-100 g of cells were suspended in 200 mL cell resuspension buffer (50 mM potassium phosphate buffer pH 7.5, 5% glycerol, 2 mM EDTA) and were lysed by 12 × 30 s bead beating cycles, with 30 s on ice between each cycle. The cell lysate was centrifuged at 10,000 x g for 40 minutes to remove unbroken cells and cell debris, after which membranes were isolated from the supernatant by ultracentrifugation at 100,000 x g for 1 hour. The membranes were homogenized and washed twice, first using wash buffer (5 mM Tris-HCl pH 9.5, 4M urea, 2 mM EDTA) followed by membrane buffer (20 mM Tris-HCl pH 8, 20 mM NaCl, 10% glycerol) supplemented with 1 mM PMSF and 2 mM EDTA. The membranes were finally suspended in membrane buffer to a final concentration of 0.5 g/ml and stored at −80°C until further use. Membranes were diluted 1:1 with solubilization buffer (20 mM Tris-HCl pH 8, 300 mM NaCl, 8% octyl-β-D-glucoside (OG, Anatrace, Maumee, OH, USA) supplemented with one cOmplete™ EDTA-free protease inhibitor cocktail tablet (Roche, Welwyn Garden City, UK) with continuous stirring for 2.5 hours at 4°C. The final solubilization volume and detergent concentration were 50 mL and 4%, respectively. Unsolubilized material was pelleted at 100,000 x g for 30 min and the supernatant was loaded on Ni-affinity column (HisTrap, GE Healthcare, Herefordshire, UK) equilibrated with AQP4 buffer (20 mM Tris-HCl pH 8, 300 mM NaCl, 10% glycerol, 1% OG) supplemented with 10 mM imidazole. After washing with AQP4 buffer containing 75 mM imidazole, AQP4 was eluted using 300 mM imidazole. Fractions were analyzed using SDS-PAGE, pooled and concentrated using a Vivaspin concentrator with 50 kDa cut-off. The concentrated sample was loaded on a Superdex 200 10/300 GL (GE Healthcare, Herefordshire, UK) equilibrated with the AQP4 buffer. After SDS-PAGE analysis, relevant fractions were pooled and concentrated as above.

A fragment of human AQP4 encoding the cytosolic carboxy-terminal tail (residues 254-323; NP_001641.1) was amplified by standard PCR techniques and sub-cloned into the pGEX-6P1 vector. The construct was verified by DNA sequencing. The GST-AQP4ct fusion protein (GST-recAQP4ct) was produced in *E. coli* strain BL21(DE3) in LB medium. GST-recAQP4ct was isolated using glutathione-Sepharose 4B resin. The phosphorylation of GST-recAQP4ct was analyzed by phosphate-binding-tag SDS-PAGE. Samples were resolved in SDS gels containing 12% acrylamide, 40 μM Phos-tag reagent (NARD Chemicals; Kobe City, Japan) and 0.1 mM MnCl_2_ at 20 mA/gel. Separated proteins were visualized with Coomassie Brilliant-Blue staining.

#### Proteoliposome shrinking assay

To evaluate whether purified, recombinant AQP4 is functionally active, we characterized water transport using a proteoliposome assay. Following purification, AQP4 was reconstituted in liposomes with a lipid composition of 1-palmitoyl-2-oleoyl-sn-glycero-3-phosphocholine (POPC), 2-oleoyl-1-palmitoyl-sn-glycero-3-phospho-rac-(1-glycerol) (POPG) and cholesterol in a 2:1:2 ratio (Tong et al., 2012). Specifically, POPC, POPG and cholesterol (Sigma) were mixed in a 2:1:2 ratio and dissolved in chloroform in a glass vial to a concentration of 25 mg/ml followed by dehydration using N_2_ forming a thin lipid bilayer. Upon dehydration, the lipid film was kept under a light N_2_ stream for 2 hours to achieve complete chloroform removal. The lipid film was rehydrated with reconstitution buffer (20mM HEPES pH 8.0, 200 mM NaCl) with added fluorophore, 10 mM (5)6-carboxyfluorescein (Sigma), to a concentration of 20 mg/ml lipids. The lipid suspension was sonicated in a sonication bath for 3 × 15 min, with a 5 min break between cycles. The lipids were frozen in liquid nitrogen and thawed three times. When thawed for the third time, the lipids were passed through a 100 nm polycarbonate filter 11 times, using an extruder (Mini-Extruder, Avanti). The lipids were diluted to 4 mg ml^-1^ with reconstitution buffer containing 25% glycerol and 1% OG, after which 0.02% Triton X-100 was added to the sample to a final concentration of 0.02%. AQP4 was added to the lipid suspension using a lipid-to-protein-ratio (LPR) of 200 and each sample was dialyzed overnight at 4°C in reconstitution buffer. The samples were centrifuged at 57,000 × g (1.5 hours) and the resulting pellets were suspended in reconstitution buffer.

The shrinkage assay was performed on an SX-20 Stopped-Flow Spectrometer system (Applied Photophysics), where the liposomes were mixed with reaction buffer with 200 mOsm sucrose. Data were collected at 495 nm at a 90° angle for 2 s. All data were collected at 18°C. Empty liposomes were used as a negative control. The data were analyzed and plotted in Pro-Data Viewer (Applied Photosystems). Data for each sample were the average of 10 readings. Data were fitted using a double exponential fit. The smallest rate constant is unaffected by changes in AQP4 reconstitution efficiency, while the larger rate constant corresponds liposomes containing AQP4. This rate constant (k) was used to calculate the osmotic water permeability, P_f_ (cm/s) = k / ((S/V_0_)^∗^V_w_^∗^C_out_), where (S/V_0_) is the initial surface area to volume ratio of the liposome, V_W_ is the partial molar volume of water (18 cm^3^ mol^-1^), and C_out_ is the external osmolality (0.1 Osm). The reconstitution experiments were performed three times to achieve data reproducibility and statistically evaluated using a t test (p < 0.05). The single-channel unit permeabilities (P_u_) were calculated using these P_f_ values divided by the AQP4 density per unit surface area (SuD) (Werten et al., 2001); P_u_ = P_f_/SuD. SuD was calculated using the theoretical area per lipid molecule value (Am; 0.47 nm^2^) (Tong et al., 2012) and an LPR of 200.

#### Labeling of human CaM

Human CaM carrying a S17C mutation was a gift from Professor Sara Linse, Lund University (O’Connell et al., 2010). As the protein lacks cysteines, the S17C mutation introduces a unique site for labeling on the opposite side of the binding cleft that is not likely to interfere with binding. CaM was labeled with Alexa Fluor 488 in a 2:1 Alexa:CaM ratio in 20 mM phosphate buffer pH 8 at room temperature (3 hours).

#### Microscale thermophoresis

MST experiments were carried out in premium coated capillaries on a Monolith NT.115 (Nanotemper Technologies, Munich, Germany). All experiments were carried out in triplicate. AQP4 constructs were diluted in a 2:1 dilution series with 20 mM Tris pH 8, 300 mM NaCl, 5 mM CaCl_2_ 1% OG and mixed 1:1 with a solution containing 32 or 50 nM CaM-Alexa Fluor 488. For experiments with EDTA and TFP, these were added to the buffer at concentrations of 10 mM and 2 mM respectively, resulting in a final concentration in the capillaries of 5 and 1 mM. For determining the affinity with wild-type AQP4, AQP4-S256E, AQP4-ΔS256 or AQP4-F258/262/266A, labeled CaM was mixed with unlabelled CaM to a final total CaM concentration of 17 μM. The mix between labeled and unlabelled CaM was important in order to avoid loss of fluorescence due to CaM-Alexa Fluor 488 sticking to the capillaries. For each AQP4 construct, three individual dilution series were prepared, each containing 12-16 samples with AQP4 concentrations ranging between 0.4 and 160 μM (wild-type AQP4), 1.0 and 200 μM (EDTA and TFP), 0.52 and 230 μM (AQP4-S276E and ΔS256) and 1.5 and 130 μM (AQP4-F258/262/266A). The samples were transferred to capillaries and MST data were obtained using MST and LED power settings of 20% and 10% (wild-type AQP4), 20% and 20% (EDTA, TFP and AQP4-F258/262/266A) and 40% and 10% (AQP4-S276E and ΔS256). F_norm_ was defined as the Fo/F1 ratio of normalized fluorescence where Fo and F1 correspond to fluorescence before and after heating, respectively (Figure S2). The time point taken as F1 was determined by the M.O. Affinity Analysis software (Nanotemper Technologies) as the time interval that gives the best signal to noise ratio. For studying the concentration-dependence of the TFP-inhibition, three separate two-fold dilution series of TFP (0.3-3 mM) were made, resulting in 12 samples each that were mixed 1:1 with a solution containing 400 μM full-length AQP4 and 64 nM CaM- AlexaFluor 488. MST-data were obtained as above using an MST and LED power setting of 80% and 20%, respectively.

#### Microscale thermophoresis data analysis

Raw data treatment was done in MO. control software (NanoTemper Technologies) and curve fitting was done using Origin (OriginLab, Northampton, MA, USA). The binding curve data could be described by a one-to-one binding model:

$$y=S1+\left(S2-S1\right)\left(\frac{L_{Free}}{L_{Free}+K_{D}}\right)$$

$$L_{Free}=0.5\left(L_{Tot}-P_{Tot}-K_{D}\right)+\sqrt{0.25{\left(K_{D}+P_{Tot}-L_{Tot}\right)}^{2}+L_{Tot}K_{D}}$$

where S1 and S2 are the signals of the unbound and bound form respectively. L_Free_ is the free monomeric [AQP4] and L_Tot_ the total monomeric [AQP4]. P_Tot_ is the total [CaM-Alexa Fluor 488] and K_d_ the dissociation constant.

The dose-response curve could be described by the following equation:

$$y=A1+\frac{A2-A1}{1+10^{\left(LOGx0-x\right)p\;}}$$

where A1 and A2 are the signals of the unbound and bound form respectively, x0 is the midpoint of the slope and p is the Hill coefficient.

#### AQP4 plasmids for expression in HEK293 cells

An AQP4-His_6_ construct for mammalian expression was generated by site-directed mutagenesis using our pDEST47-hAQP4-GFP plasmid (Kitchen et al., 2015) as template. The first 8 codons of the GFP tag were mutated to 6 alternating histidine codons (CAC/CAT), followed by two in-frame stop codons. This plasmid was used as template for site-directed mutagenesis to make AQP4-F258A-His_6_, AQP4-F262A-His_6_, AQP4-F266A-His_6_, and AQP4-F258/262/266A-His_6_.

#### Immobilised metal affinity chromatography to capture AQP4-CaM complexes

To measure endogenous calmodulin binding to AQP4-6xHis_6_ or AQP4-F258/262/266A-His_6_ in HEK293 cells, cells were transiently transfected for 24 hours, then exposed to a fourfold reduction of extracellular osmolarity for 10 minutes to induce cell swelling. Cells were lysed using 1% Triton X-100, 150 mM NaCl, 2 mM CaCl_2_, 25 mM Tris pH 7.4, supplemented with fresh EDTA-free protease inhibitor cocktail (Sigma-Aldrich, 11873580001). Insoluble material was removed by centrifugation at 20,000 g for 10 minutes, and His_6_-tagged proteins and their interacting partners were precipitated using HisPur Ni-NTA magnetic beads (ThermoFisher, 88831) following the manufacturer’s instructions. 75 mM imidazole was used for washing and 300 mM for elution. All buffers were supplemented with 2 mM CaCl_2_.

#### NMR experiments

Uniformly ^13^C, ^15^N-labeled GST-recAQP4ct was prepared in M9 minimal medium containing 0.5 g/L ^15^NH_4_Cl and 1 g/L ^13^C-glucose (Cambridge Isotope Laboratories), captured on glutathione-Sepharose 4B resin and cleaved on-column by treatment with PreScission Protease. The eluted recAQP4ct protein contained the cloning artifact ‘GPLGS’ at its amino-terminus. The ^15^N, ^13^C-rAQP4ct protein was concentrated and exchanged into buffers for NMR with an Amicon centrifugal filter (Millipore). Concentration was determined using its predicted molar extinction coefficient (1490 cm^-1^ M^-1^ at 280nm). NMR samples of recAQP4ct contained 0.5 mM ^13^C, ^15^N-labeled recAQP4ct, 20 mM Bis-Tris (pH 7), 0.03% NaN_3_, 100 mM KCl, 6 mM CaCl_2_ and 0.5 mM 2,2-dimethyl-2-silapentane-5-sulfonate (DSS) in 90% H_2_O/10% D_2_O. NMR samples of recAQP4ct complexed with CaM contained an additional 1 mM CaM (Nakashima et al., 1996). All NMR experiments were performed at 30°C on a Bruker Avance 600 MHz NMR spectrometer. Sequential assignments of the main-chain atoms of recAQP4ct with and without CaM were achieved using two-dimensional ^1^H, ^15^N-HSQC and three-dimensional HNCACB, CBCA(CO)NH, HNCO, HN(CA)CO, HNCA, and HN(CO)CA experiments. All NMR spectra were processed with NMRPipe and analyzed using NMRViewJ (Delaglio et al., 1995, Johnson, 2004).

#### Analysis of spinal water content

Immediately after sacrificing rats, spinal cord tissue was dissected 5 mm either side of the lesion site. Tissue was then weighed in Eppendorf tubes, dried at 95°C for 48 hours and reweighed. The percent water content was calculated as water content (%) = [(wet weight-dry weight)/ wet weight] × 100% (Li and Tator, 1999).

#### Immunohistochemistry

Rats were culled by rising concentrations of CO_2_ in accordance with UK Home Office Animals (Scientific Procedures) Act 1986. Animals were then intracardially perfused with 4% paraformaldehyde in phosphate-buffered saline (PBS). Spinal cords were dissected ± 5 mm from the lesion site and cryo-protected in increasing sucrose concentrations in PBS, before being embedded in OCT Compound (ThermoFisher Scientific) and snap frozen on dry ice. Tissue was sectioned onto Superfrost Plus Slides (ThermoFisher Scientific, Loughborough, UK) at 30 μm using a Bright cryostat (Bright Instrument, Cambridgeshire, UK) through the parasagittal plane of the cord and stored at −20°C until required. To detect AQP4, Foxo3 and RECA-1, sections were defrosted, blocked with normal goat and rat serum (ThermoFisher Scientific, Cat No. 01-6201 and 10710C) at 3% each in 0.3% PBS-Tween20 and incubated overnight at 4°C in antibody-diluting buffer containing 1:400 rabbit anti-AQP4 IgG (Abcam, Cat No. ab128906) or 1:200 rabbit anti-Foxo3 IgG (Abcam, Cat No. ab23683) and 1:50 mouse anti-RECA-1 (Abcam, Cat No. ab9774). Sections were then washed in PBS and incubated in 1:400 secondary antibody goat anti-rabbit IgG FITC, (Merck KGaA, Darmstadt, DE, Cat No. F0382) and/or 1:400 goat anti-mouse IgG Alexafluor 633 (ThermoFisher Scientific) for 2 hours at room temperature before being washed again and mounted with Fluoroshield with DAPI (Merck KGaA). Immunohistochemistry for albumin was performed as above, but with the blocking buffer step after the primary antibody incubation of 1:100 chicken anti-albumin (Abcam, Cat No. ab106582) followed by 1:200 secondary goat anti-chicken IgY Alexafluor 488 (Abcam, Cat No. ab150169). To detect laminin, frozen sections were allowed to thaw for 30 minutes at room temperature and blocked in 3% H_2_O_2_ and 5% normal goat serum, followed by incubation with 1:100 rabbit anti-laminin (Sigma, Cat No. L9393) overnight at 4°C. This was followed by incubation with biotinylated linking antibody and HRP (Dako), with brief rinses in PBS between incubations. The reaction was visualized using 3,3′-diaminobenzidine (Vector Laboratories) and counterstained with Mayer’s hematoxylin. Reagent controls (omitting the primary antibody or substituting non-immune serum for the primary antibody) on tissue sections revealed no staining, thus confirming the specificity of the primary antibodies used.

#### Image acquisition and analysis of total and relative AQP4 levels

Imaging was done using an upright Leica Microsystems SP5 TCS II with multiphoton add-on and a 63x magnification apochromatically-corrected (APO) oil lens. Laser properties were 488 Argon @ 20% (anti AQP4 – AlexaFluor 488; green); 633 HeNe laser at 29% (anti RECA1 – AlexaFluor 633; red); 800 nm multiphoton laser for DAPI (SpectraPhysics MaiTai; blue). X, Y resolution was 1,024 × 1,024 pixels for all images. The spectral range collected for AQP4 was 499-570 nm and RECA1 was 642-785 nm. Laser power, pixel size, scanning speed, smart gain and offset and AOBS settings were kept constant for analysis of total AQP4 levels. To facilitate accurate acquisition and analysis of relative astrocytic endfoot to process levels of AQP4, smart gain and offset were adjusted using the quick LUT, glow over and under setting, to create a dynamic fluorescence range (0-255). Images were analyzed using ImageJ. The relative intensity of BSCB (peri-endothelial) AQP4 signal was determined by drawing 2 dimensional ROIs along peri-endothelial endfeet or along astrocyte processes away from the BSCB (3-10 ROIs per image, depending on the number of endothelial cells in the image). The ratio of intensity (peri-endothelial/non-peri-endothelial) was calculated per image (4-9 images per animal), and averaged for each animal. For the analysis of total AQP4 levels, ROIs were the entire original field of view.

#### Quantification of albumin immunostaining and cavity size

To eliminate sample bias caused by variation in the size of the wound cavity at different wound depths, cavity sizes in sections taken from similar depths in each animal were compared and quantified (Esmaeili et al., 2014). Briefly, laminin immunolabeling was performed at defined depths through the lesion site and the cavity size was quantified using Image-Pro Analyzer 6.2 (Media Cybernetics Inc., Bethesda, MD, USA) after setting the background threshold levels using antibody control sections (omission of primary antibody). Cavity size was determined by tracing around the cavity perimeter and determining the area (n = 12 biological repeats). For albumin immunolabeling, 3 sections from defined depths through the cord from each treatment group were analyzed. Albumin was quantified by cropping images to defined ROIs containing the lesion site and auto-thresholding to remove background pixels (auto-threshold was set at a level where antibody control sections were counted at 0 pixels). The number of pixels with an intensity above the background threshold was counted (n = 3 biological repeats).

#### Analysis of brain water content

Immediately after sacrificing rats, 5 mm of coronal brain tissue centered around the lesion site was removed from the ipsilateral cortex (or corresponding contralateral cortex (as controls)), weighed on aluminum foil, dried at 65°C for 24 hours and re-weighed. The percent water content was calculated as water content (%) = [(wet weight-dry weight)/wet weight] x 100% (Dempsey et al., 2000).

#### Protein extraction, immunoblot and densitometry

Total protein from DRG or spinal cord tissues was extracted, immunoblotted and analyzed by densitometry. Briefly, 40 μg of total protein was resolved on 12% SDS gels, transferred to polyvinylidene fluoride (PVDF) membranes (Millipore, Watford, UK) and probed with relevant primary antibodies: anti-AQP4 (Abcam, Cambridge UK, Cat No. ab46182; 1:400 dilution) and β-actin (Sigma, Cat no A2228.1:1000 dilution). Membranes were then incubated with relevant HRP-labeled secondary antibodies and bands were detected using an enhanced chemiluminescence kit (all from GE Healthcare, Buckinghamshire, UK). Anti β-actin antibodies were used as a protein loading control. For densitometry, blots were scanned into Adobe Photoshop (Adobe Systems Inc, San Jose, CA, USA) keeping all scanning parameters the same between blots and the integrated density of the bands was analyzed using the built-in macros for gel analysis in ImageJ (NIH, USA, https://imagej.nih.gov/ij). Means ± SEM were plotted in Microsoft Excel (Microsoft Corporation, CA, USA). For cultured cells, 10 μg protein was run on hand-cast 8% acrylamide Tris-glycine SDS-PAGE gels using a Tris-glycine-SDS running buffer. Proteins were transferred to 0.2 μm PVDF using a Tris-glycine-methanol transfer buffer. For immunoblotting of CaM, 16% gels were used and 2 mM CaCl_2_ was added to the standard Tris-glycine-methanol transfer buffer (McKeon and Lyman, 1991).

#### Electrophysiology after CNS edema

Six weeks after DC crush injury and treatment, compound action potentials (CAP) were recorded after vehicle, CaM_i_ (TFP), PKAi (H89) or PKCi (Gö 6983) treatment. Briefly, with the experimenter masked to the treatment conditions, animals were deeply anesthetized using 5% isoflurane and deep anesthesia maintained with 1.5% isoflurane for the duration of the experiment. Heart rate was carefully monitored and body temperature maintained using a feedback-controlled thermal blanket. Pancuronium bromide (0.3 mg/kg, Sigma) was injected intraperitoneally to minimize muscular contractions that may interfere with the electrophysiological assessments. A Kopf stereotaxic apparatus (Kopf Instruments, Tujunga, CA, USA) was used to affix rats into position and a midline incision made through the skin, followed by laminectomy and exposure of the thoracic and lumbar spinal regions. The dura was cut and the exposed spinal cord was bathed in warm mineral oil. Silver wire electrodes (0.01 inch diameter; A-M Systems, Carlsborg, WA, USA) that were insulated except at the tip were used to stimulate the DC axons at L1/2 and CAPs were recorded at C4/5 along the midline surface of the spinal cord. Stimulating single current pulses (0.05 ms) were applied in increasing increments at L1/2 (0.2, 0.3, 0.6, 0.8 and 1.1 mA) with the signal from the recording electrode amplified with filters set at 300-3,000 Hz, collected and analyzed using Spike 2 software (Cambridge Electronic Design, Cambridge, UK). At the end of the experiment, the dorsal half of the spinal cord was completely transected between stimulating and recording electrodes to confirm that a CAP could not be detected. Finally, with the animals under deep anesthesia, animals were killed by overdose of anesthetics. CAP amplitudes were calculated between the negative deflection after the stimulus artifact and the next peak of the wave. CAP area was calculated by rectifying the negative component (full-wave rectification in Spike 2 software) and measuring its area at the different stimulation intensities. Superimposed Spike 2 software processed data are shown for representative CAP traces (Almutiri et al., 2018).

#### Functional tests after CNS edema

Functional testing was carried out after injury (Almutiri et al., 2018, Fagoe et al., 2016). Briefly, masked and randomly-assigned animals (n = 18/group/test) received training to master traversing a horizontal ladder for 1 week before functional testing. Baseline parameters for all functional tests were established 2-3 days before injury. Animals were then tested 2 days after DC lesion + treatment and then weekly for 6 weeks. Experiments were performed by an observer blinded to treatment with animals tested in the same order and at the same time of day. Three individual trials were performed each time for each animal.

##### Horizontal ladder crossing test

This tested the animals’ locomotor function and was performed on a 0.9-m-long horizontal ladder with a diameter of 15.5 cm and randomly adjusted rungs with variable gaps of 3.5-5.0 cm. Animals were assessed traversing the ladder with the total number of steps taken to cross the ladder and the number of left and right rear paw slips being recorded. The mean error rate was then calculated by dividing the number of slips by the total number of steps taken.

##### Tape sensing and removal test (sensory function)

The tape sensing and removal test determines touch perception from the left hind paw. Animals were held with both hind-paws extended and the time it took for the animal to detect and remove a 15x15 mm piece of tape (Kip Hochkrepp, Bocholt, Germany) was recorded and used to calculate the mean sensing time.

### Quantification and Statistical Analysis

All statistical details for the experiments performed can be found in the figure legends, including the statistical tests used, the exact value of n, what n represents and the precision measures. For *in vitro* data that were normally distributed, ANOVA with a Bonferroni post hoc test was used to determine statistical differences between means. The data in Figure 1F are presented as a fold-change normalized to the experimental control. ELISA and cell-surface biotinylation data were found to be nonparametric in distribution using the Shapiro-Wilk test, so a Kruskall-Wallis analysis with a Conover-Inman post hoc test was used to identify significant differences (p ≤ 0.05) using StatsDirect 3 software. For *in vivo* data, no statistical methods were used to predetermine sample sizes based on previous studies of a similar nature (Almutiri et al., 2018).

#### Analysis of functional tests

the complete time-course of lesioned and sham-treated animals for the horizontal ladder crossing test was compared using binomial (since individual steps are scored as a successful step or a slip, therefore following a binomial distribution) generalized linear mixed models (GLMM) (Fagoe et al., 2016). Animals were set as random factors, time as a continuous covariate, lesioned/sham (‘LESION’) was set to ‘true’ in lesioned animals, ‘false’ otherwise and operated/unoperated (‘OPERATED’) set to ‘false’ before surgery and ‘true’ after surgery, as fixed factors. Binomial GLMMs were fitted in R using package *lme4* with the *glmer* functions set using the following model formulae:

(Model 1) outcome∼LESION∗time+OPERATED+(time∖animal)

(Model 2) outcome∼LESION+time+OPERATED+(time∖animal)

(Model 3) outcome∼LESION+time+OPERATED+(1∖animal)

(Model 4) outcome∼time+OPERATED+(1∖animal)

Bracketed terms refer to the ‘random effects’ and account for repeated-measurements in estimation of the effect sizes and significant of INT (Interaction term of LESION over time, i.e., Model 1) and LESION. P values for GLMMs were calculated by model comparison using parametric bootstrap for INT and LESION against the null hypothesis that each parameter is zero, using *pbkrtest* in R package with 1,000 and 20,000 simulations.

#### Analysis of tape sensing removal test

the time-courses of lesioned versus sham animals were compared using linear mixed models (LMMs) with the R package *lme4* with the *glmer* functions set with the same model formulae as for the ladder crossing tests. Standard regression diagnostics (quantile plots of the residuals versus the normal distribution, plots of residuals versus fitted values) were carried out for the data fitted with LMMs and P values for the INT and LESION parameters of the LMMs were calculated by model comparison using package *pbkrtest* in R, with the Kenward-Roger method (Fagoe et al., 2016). Independent sample t tests were performed in SPSS, Version 21 (IBM). For testing of the significance between binding constants determined by MST, a Z-test for two population means was used:

$$Z=\frac{\left(\bar{X_{1}}-\bar{X_{2}}\right)}{\left(\sigma_{1}^{2}+\sigma_{2}^{2}\right)}$$

where X_1_ and X_2_ are the two binding constants, σ_1_ and σ_2_ their standard deviation.

### Data and Code Availability

The published article contains all datasets generated and analyzed during this study.

## Supplemental Information

Table S1. Mean ± SEM CAP Response Latencies after DC Injury and Vehicle, CaM_i_, PKA_i_, or PKC_i_ Treatment, Related to Figure 4 Despite CAP response latencies in DC + CaM_i_ and PKA_i_-treated rats being approximately 3 times longer than Sham-rats, no CAP response latencies were detectable in DC + Vehicle or DC + PKC_i_-treated rats due to the absence of evoked activity. ND = not detectable.

Table S2. p Values for Statistical Comparisons, Related to Figures 1, 2, 4, and S1–S6 p values highlighted in bold text have values < 0.05.

Figure360. An Author Presentation of Figure 5
