# Supplementary material for: Targeting Aquaporin-4 Subcellular Localization to Treat Central Nervous System Edema
Source: Cell. 2020 May 14;181(4):784–799.e19. doi: 10.1016/j.cell.2020.03.037 (PMC7242911; doi:10.1016/j.cell.2020.03.037)
Supplement: Table S1. Mean ± SEM CAP Response Latencies after DC Injury and Vehicle, CaMi, PKAi, or PKCi Treatment, Related to Figure 4 — Despite CAP response latencies in DC + CaMi and PKAi-treated rats being approximately 3 times longer than Sham-rats, no CAP response latencies were detectable in DC + Vehicle or DC + PKCi-treated rats due to the absence of evoked activity. ND = not detectable. [file mmc1.pdf]

Cell, Volume *181*

## Supplemental Information

# Targeting Aquaporin-4 Subcellular Localization

# to Treat Central Nervous System Edema

**Philip Kitchen, Mootaz M. Salman, Andrea M. Halsey, Charlotte Clarke-Bland, Justin A. MacDonald, Hiroaki Ishida, Hans J. Vogel, Sharif Almutiri, Ann Logan, Stefan Kreida, Tamim Al-Jubair, Julie Winkel Missel, Pontus Gourdon, Susanna Törnroth-Horsefield, Matthew T. Conner, Zubair Ahmed, Alex C. Conner, and Roslyn M. Bill**

**Supplementary Table S1.** Mean ± SEM CAP response latencies after DC injury and Vehicle, $CaM_i$, $PKA_i$ or $PKC_i$ treatment. Despite CAP response latencies in DC + $CaM_i$ and $PKA_i$-treated rats being approximately 3 times longer than Sham-rats, no CAP response latencies were detectable in DC + Vehicle or DC + $PKC_i$-treated rats due to the absence of evoked activity. ND = not detectable. Related to Figure 4.

| | **Sham** | **DC + Vehicle** | **DC + $CaM_i$** | **DC + $PKA_i$** | **DC + $PKC_i$** |
|---|---|---|---|---|---|
| Mean latency ± SEM (ms) | 6.2 ± 1.5 | ND | 20.6 ± 4.9 | 20.4 ± 4.8 | ND |
