# Supplementary material for: Targeting Aquaporin-4 Subcellular Localization to Treat Central Nervous System Edema
Source: Cell. 2020 May 14;181(4):784–799.e19. doi: 10.1016/j.cell.2020.03.037 (PMC7242911; doi:10.1016/j.cell.2020.03.037)
Supplement: Table S2. p Values for Statistical Comparisons, Related to Figures 1, 2, 4, and S1–S6 — p values highlighted in bold text have values < 0.05. [file mmc2.pdf]

Supplementary Table S2. p values for statistical comparisons in Figures 1, 2, 4 and S1-6; p values highlighted in bold text have values < 0.05.

Figure	Panel	Comparison	p/corrected p
**1**	**a**	Hypoxia *vs* control	**0.00044**
		Hypotonicity *vs* control	**0.046**
		EGTA-AM *vs* control	11
		Hypoxia EGTA-AM *vs* control	6.4
		Hypotonicity EGTA-AM *vs* control	0.43
		TFP *vs* control	8.8
		Hypoxia TFP *vs* control	7.9
		Hypotonicity TFP *vs* control	2.1
		$TRPV4_i$ *vs* control	11
		Hypoxia $TRPV4_i$ *vs* control	4.4
		Hypotonicity $TRPV4_i$ *vs* control	13
		$TRPV4_a$ *vs* control	**0.0016**
		Hypoxia $TRPV4_a$ *vs* control	**0.00024**
		Hypotonicity $TRPV4_a$ *vs* control	**2.5×10^{-5}**
	**b**	1 h *vs* normoxia	0.25
		3 h *vs* normoxia	0.96
		6 h *vs* normoxia	**0.035**
		6+1 h *vs* normoxia	**0.0036**
		6+3 h *vs* normoxia	**0.041**
		6+6 h *vs* normoxia	0.15
	**c**	TFP *vs* normoxia	0.074
		EGTA-AM *vs* normoxia	0.056
		Hypoxia *vs* normoxia	**0.015**
		Hypoxia TFP *vs* normoxia	0.96
		Hypoxia EGTA-AM *vs* normoxia	0.13
	**d**	Hypoxia *vs* normoxia RM 2-way ANOVA hypoxia×time interaction	**0.00070**
	**f**	cAMP control *vs* forskolin	**0.018**
		cAMP control *vs* hypotonicity	**0.015**
		cAMP control *vs* hypotonicity Ca^{2+} free	1.2
		cAMP control *vs* hypotonicity + W-7	0.67
		cAMP control *vs* hypotonicity + TFP	0.20
		cAMP control *vs* CALP-3	**0.028**
		PKA control *vs* forskolin	**9.1×10^{-5}**
		PKA control *vs* hypotonicity	**0.0023**
		PKA control *vs* hypotonicity Ca^{2+} free	0.99
		PKA control *vs* hypotonicity + W-7	0.80
		PKA control *vs* hypotonicity + TFP	3.3
		PKA control *vs* CALP-3	**0.0024**
**2**	**a**	3 day sham *vs* DC+Vehicle	**3.7×10^{-5}**
		3 day sham *vs* DC+CaMi	**0.0060**
		3 day sham *vs* DC+PKAi	**0.0050**
		7 day sham *vs* DC+Vehicle	**0.024**
		7 day sham *vs* DC+CaMi	**0.021**
		7 day sham *vs* DC+PKAi	**0.019**
		28 day sham *vs* DC+Vehicle	0.56
		28 day sham *vs* DC+CaMi	0.16
		28 day sham *vs* DC+PKAi	1.0
	**b**	Sham *vs* DC+Vehicle	**5.0×10^{-6}**
		Vehicle *vs* TFP	**0.032**
		Vehicle *vs* W-7	**4.3×10^{-5}**
		Vehicle *vs* PKA_i	**0.00029**
		Vehicle *vs* $AR1_i$	0.75
		Vehicle *vs* $D2R_i$	**0.038**
		Vehicle *vs* PKC_i	**0.038**
	**g**	Sham *vs* DC+Vehicle	**3.2×10^{-7}**
		Vehicle *vs* CaM_i	**1.3×10^{-6}**
		Vehicle *vs* PKA_i	**9.0×10^{-6}**
	**h**	Sham *vs* DC+Vehicle	**0.0020**
		Vehicle *vs* CaMi	**0.022**
		Vehicle *vs* PKAi	**0.0090**

**4**	**b**	Vehicle *vs* CaM_i<br>Vehicle *vs* PKA_i<br>Vehicle *vs* PKC_i	**0.00012**<br>**0.00011**<br>3.8
	**c**	Vehicle *vs* CaM_i<br>Vehicle *vs* PKA_i<br>Vehicle *vs* PKC_i	**0.00013**<br>**0.00014**<br>2.1
	**d**	Sham *vs* DC+Vehicle<br>Vehicle *vs* CaM_i<br>Vehicle *vs* PKA_i<br>Vehicle *vs* PKC_i	**0.00010**<br>**0.00010**<br>**0.00011**<br>4.2
	**e**	Sham *vs* DC+Vehicle<br>Vehicle *vs* CaM_i<br>Vehicle *vs* PKA_i<br>Vehicle *vs* PKC_i	**0.0010**<br>**0.0013**<br>**0.0014**<br>1.8
	**f**	Sham *vs* DC+Vehicle<br>Vehicle *vs* CaM_i<br>Vehicle *vs* PKA_i	$\mathbf{6.0 \times 10^{-5}}$<br>**0.0015**<br>**0.00089**
	**g**	DC+Vehicle *vs* DC+CaM_i	$\mathbf{4.0 \times 10^{-10}}$
**S1**	**c**	Control *vs* shAQP4 3 dpi<br>Control *vs* shAQP4 28 dpi	**0.00012**<br>$\mathbf{9.0 \times 10^{-5}}$
	**d**	Vehicle *vs* control 3dpi<br>Vehicle *vs* shAQP4 3dpi<br>Vehicle *vs* control 28 dpi<br>Vehicle *vs* shAQP4 28 dpi	4.4<br>**0.00011**<br>4.2<br>$\mathbf{9.0 \times 10^{-5}}$
**S2**		Sham *vs* injury contra<br>Injury *vs* injury+CaM_i ipsi<br>Injury *vs* injury+PKA_i ipsi<br>Injury *vs* injury+PKC_i ipsi<br>Sham *vs* injury ipsi<br>Injury *vs* injury+CaM_i contra<br>Injury *vs* injury+PKA_i contra<br>Injury *vs* injury+PKC_i contra	**0.00011**<br>**0.00010**<br>**0.00011**<br>2.3<br>2.3<br>3.1<br>2.8<br>2.8
**S3**	**d**	Sham *vs* DC+Vehicle<br>Sham *vs* DC+Vehicle (away)<br>DC+Vehicle *vs* DC+Vehicle (away)	$\mathbf{2.9 \times 10^{-5}}$<br>1.0<br>**0.00010**
	**e**	Sham *vs* DC+Vehicle<br>Sham *vs* DC+Vehicle (away)<br>DC+Vehicle *vs* DC+Vehicle (away)	**0.0080**<br>1.0<br>**0.010**
**S4**	**e**<br><br><br><br><br><br>**h**	PKA control *vs* forskolin<br>PKA control *vs* hypotonicity<br>PKA control *vs* hypotonicity+TFP<br>Akt control *vs* forskolin<br>Akt control *vs* hypotonicity<br>Akt control *vs* hypotonicity+TFP<br>Nuclear FOXO3A control *vs* forskolin 10 min<br>Nuclear FOXO3A control *vs* hypotonicity 10 min<br>Nuclear FOXO3A control *vs* forskolin 1 h<br>Nuclear FOXO3A control *vs* hypotonicity 1 h<br>Nuclear FOXO3A control *vs* forskolin 6 h<br>Nuclear FOXO3A control *vs* hypotonicity 6 h	**0.00050**<br>**0.014**<br>3.4<br>**0.038**<br>**0.044**<br>2.8<br>0.078<br>2.9<br>**0.00060**<br>0.11<br>**0.0047**<br>**0.0066**
**S5**	**g**	AQP4 translocation WT *vs* F258/262/266A<br>AQP4 translocation WT *vs* F258A<br>AQP4 translocation WT *vs* F262A<br>AQP4 translocation WT *vs* F266A	**0.00040**<br>1.4<br>3.3<br>0.72
**S6**	**b**	AQP4 *vs* control	**0.0015**
